# Functional Conservation of the Glide/Gcm Regulatory Network Controlling Glia, Hemocyte, and Tendon Cell Differentiation in *Drosophila*

**DOI:** 10.1534/genetics.115.182154

**Published:** 2015-11-12

**Authors:** Pierre B. Cattenoz, Anna Popkova, Tony D. Southall, Giuseppe Aiello, Andrea H. Brand, Angela Giangrande

**Affiliations:** * Department of Functional Genomics and Cancer, Institut de Génétique et de Biologie Moléculaire et Cellulaire, UMR7104, F-67404 Illkirch Cedex, France; † Centre National de la Recherche Scientifique (CNRS), UMR7104, F-67404 Illkirch Cedex, France; ‡ Institut National de la Santé et de la Recherche Médicale (INSERM), U964, F-67404 Illkirch Cedex, France; § Université de Strasbourg, F-67404 Illkirch, France; ** Gurdon Institute and Department of Physiology, Development and Neuroscience, University of Cambridge, United Kingdom CB2 1QN

**Keywords:** *glide/gcm*, *Drosophila*, DamID, mGcm, screen

## Abstract

High-throughput screens allow us to understand how transcription factors trigger developmental processes, including cell specification. A major challenge is identification of their binding sites because feedback loops and homeostatic interactions may mask the direct impact of those factors in transcriptome analyses. Moreover, this approach dissects the downstream signaling cascades and facilitates identification of conserved transcriptional programs. Here we show the results and the validation of a DNA adenine methyltransferase identification (DamID) genome-wide screen that identifies the direct targets of Glide/Gcm, a potent transcription factor that controls glia, hemocyte, and tendon cell differentiation in *Drosophila*. The screen identifies many genes that had not been previously associated with Glide/Gcm and highlights three major signaling pathways interacting with Glide/Gcm: Notch, Hedgehog, and JAK/STAT, which all involve feedback loops. Furthermore, the screen identifies effector molecules that are necessary for cell-cell interactions during late developmental processes and/or in ontogeny. Typically, immunoglobulin (Ig) domain–containing proteins control cell adhesion and axonal navigation. This shows that early and transiently expressed fate determinants not only control other transcription factors that, in turn, implement a specific developmental program but also directly affect late developmental events and cell function. Finally, while the mammalian genome contains two orthologous *Gcm* genes, their function has been demonstrated in vertebrate-specific tissues, placenta, and parathyroid glands, begging questions on the evolutionary conservation of the Gcm cascade in higher organisms. Here we provide the first evidence for the conservation of Gcm direct targets in humans. In sum, this work uncovers novel aspects of cell specification and sets the basis for further understanding of the role of conserved *Gcm* gene regulatory cascades.

UNDERSTANDING the molecular signature of a developmental pathway is a major challenge in modern biology. Transcription factors specify cell fates by inducing the expression of specific genes. For instance, the zinc finger transcription factor glial cell deficient/glial cell missing (Glide/Gcm, or Gcm for the sake of simplicity) is expressed transiently at early stages (Bernardoni *et al.* 1997; Laneve *et al.* 2013; Flici *et al.* 2014) and controls *Drosophila* glial and blood development (Hosoya *et al.* 1995; Jones *et al.* 1995; Vincent *et al.* 1996; Bernardoni *et al.* 1997; Egger *et al.* 2002; Freeman *et al.* 2003; Soustelle *et al.* 2004; Altenhein *et al.* 2006). Gcm is also expressed in tendon and peritracheal cells (Soustelle *et al.* 2004; Laneve *et al.* 2013), showing that fate determinants have a much broader role than expected and likely trigger the expression of target genes depending on the transcriptional and epigenetic environment of the different cell types. Expression profiling data and computational predictions were used previously to gain a better understanding of the Gcm regulatory network (Egger *et al.* 2002; Freeman *et al.* 2003; Altenhein *et al.* 2006), but these approaches did not allow genome-wide identification of the direct targets. Genes directly targeted by transcription factors are commonly identified by chromatin immunoprecipitation (CHiP) using specific antibodies targeting the transcription factors. Because no efficient antibody is available for Gcm (Popkova *et al.* 2012; Laneve *et al.* 2013), we decided to use DNA adenine methyltransferase identification (DamID) to identify the Gcm direct targets in *Drosophila*.

The DamID chromatin profiling is a methylation-based tagging method used to identify the direct genomic loci bound by transcription factors (van Steensel and Henikoff 2000; van Steensel *et al.* 2001). The approach is based on the fusion of a bacterial Dam methylase to a protein of interest to mark the factor’s genomic binding sites by adenine methylation. The DamID screen allowed us to identify 1031 targets, only some of which have already been associated with a Gcm-dependent cascade. Several targets belong to the Notch (N), JAK/STAT, and Hedgehog (Hh) pathways and suggest the presence of feedback loops. Because these pathways were previously shown to affect the cell populations depending on Gcm, the DamID data provide a molecular frame to clarify the observed mutant phenotypes (Hosoya *et al.* 1995; Jones *et al.* 1995; Bernardoni *et al.* 1997). The DamID screen also brought to light two key features of the Gcm pathway.

First, we address the late role of fate determinants beyond their ability to trigger novel transcriptional programs that are subsequently maintained by other factors [reviewed in Cattenoz and Giangrande (2015)]. The transiently expressed Gcm transcription factor is known to induce the expression of Reverse polarity (Repo), Tramtrack (Ttk), and Pointed (Pnt) transcription factors that will ensure and maintain the glial-specific differentiation program (Flici *et al.* 2014) [reviewed in Cattenoz and Giangrande (2015)], and many Gcm targets identified by the DamID screen code for transcription factors. In addition, however, we found a significantly high number of effector genes, including numerous members of the Ig domain–containing protein family. These are molecules that affect cell function or late developmental events, including cell migration, a key feature of glia and hemocytes (Schmucker *et al.* 2000; Watson *et al.* 2005; kumar *et al.* 2015) [reviewed in Schwabe *et al.* 2009)]. This suggests that early genes such as *gcm* may have a much broader impact than expected in cell specification/physiology.

Second, the Gcm pathway is conserved in evolution. The Gcm protein is structurally conserved, as are most key developmental factors present in the fly genome. Like the fly ortholog, murine mGcm1 (mGcm1) and mGcm2 are important transcription factors because their deletion is lethal (Anson-Cartwright *et al.* 2000; Gunther *et al.* 2000). However, the main role of the mammalian genes, including the human genes, is, respectively, in the placenta and the parathyroid glands, two tissues that do not exist in invertebrates (Kim *et al.* 1998; Basyuk *et al.* 1999, 2009; Gordon *et al.* 2001; Correa *et al.* 2002; Chen *et al.* 2004; Mannstadt *et al.* 2008, 2011; Doyle *et al.* 2012; Yi *et al.* 2012; Park *et al.* 2013; Mitsui *et al.* 2014). The DamID data allow us to identify direct targets that are common in flies and vertebrates. To the best of our knowledge, this is the first evidence of functional conservation and sets the basis to further understand the Gcm network in mammals.

## Materials and Methods

### DamID technique

The pUASTattB-NDam construct was made by cloning the Dam-Myc cassette from pNDam-Myc (van Steensel and Henikoff 2000; van Steensel *et al.* 2001), using *Eco*RI and *Bgl*II, into pUASTattB. To produce the Dam-Gcm fusion construct, the *gcm* full-length coding sequence was cloned into pUASTattB-NDam (Choksi *et al.*, 2006) using *Kpn*I and *Not*I sites. The two constructs were used to produce UAS Dam and UAS Dam-Gcm flies, respectively, employing the docking site *attP-22A* (Bischof *et al.* 2007). Stage 10–11 embryos [4–7 hr after egg laying (AEL)] were collected from the two strains. DNA isolation, processing, and amplification were performed as described previously (Choksi *et al.*, 2006). The Dam-only and Dam-Gcm samples were labeled and hybridized together on a whole-genome 2.1 million–feature tiling array with 50- to 75-mer oligonucleotides spaced at ∼55-bp intervals (Nimblegen Systems). Arrays were scanned and intensities extracted (Nimblegen Systems). Three biological replicates (with one dye swap) were performed. Log_2_ ratios of each spot were median normalized.

### DamID analysis

A peak-finding algorithm with false-discovery-rate (FDR) analysis was developed to identify significant binding sites (PERL script available on request). All peaks spanning eight or more consecutive probes (∼900 bp) over a twofold ratio change were assigned a FDR value. To assign a FDR value, the frequency of a range of small peak heights (0.1–1.25 log_2_ increase) were calculated within a randomized data set (for each chromosome arm) using 20 iterations for each peak size. This was repeated for a range of peak widths (6–15 consecutive probes). All these data were used to model the exponential decay of the FDR with respect to increasing peak height and peak width, therefore enabling extrapolation of FDR values for higher and broader peaks. This analysis was performed independently for each replicate data set. Each peak was assigned the highest FDR value from the three replicates. Genes were defined as targets where a binding event (with FDR < 0.1%) occurred within 5 kb of the transcriptional unit (depending on the proximity of adjacent genes).

### Conservation of the Gcm binding sites located in DamID peaks

**Figure 1:**
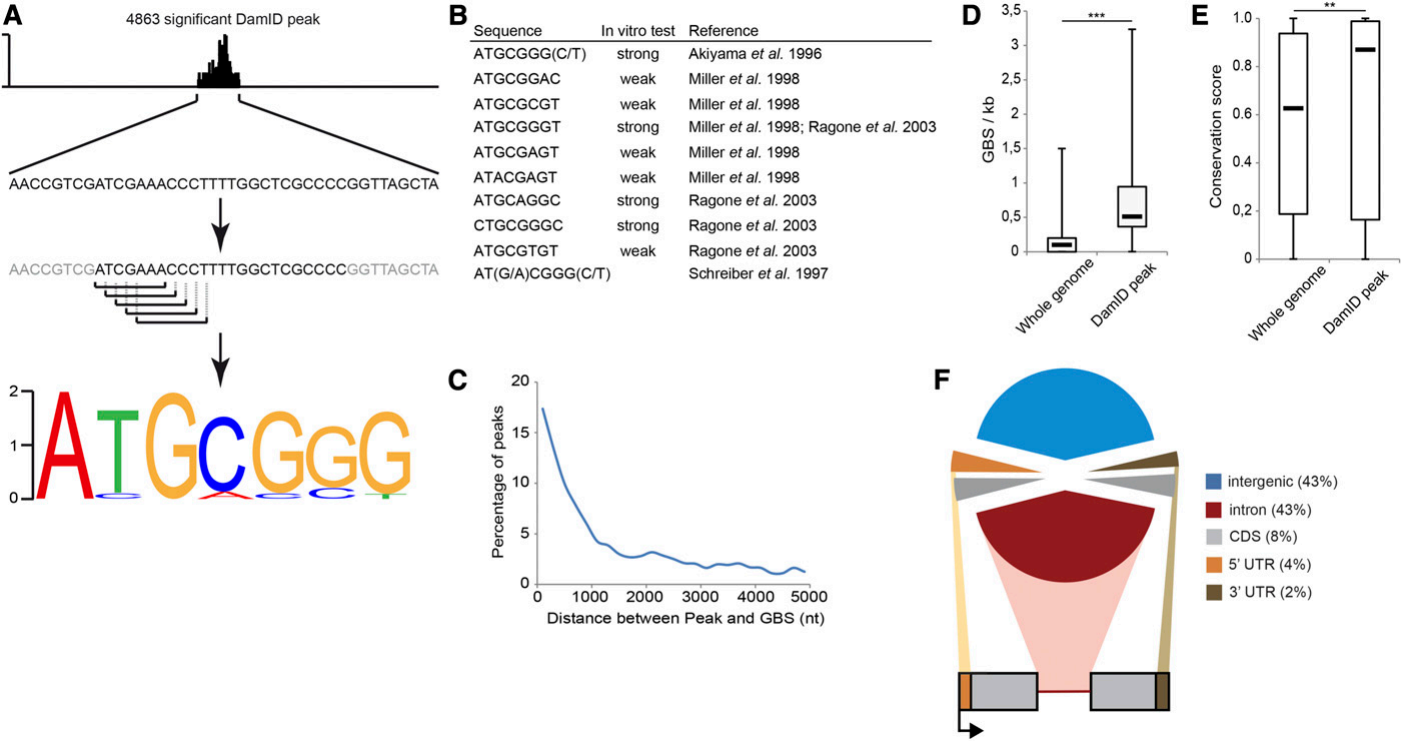
The DamID peaks are enriched for GBSs. (A) Schematic of the MICRA algorithm used to identify enriched motifs in the DamID peaks. For each peak, 1000 nt of sequence was extracted and filtered for conserved sequence, and then the frequency of every 6–10 mers was compared to the background frequency in nonexonic DNA and ranked accordingly [for details, see Southall and Brand (2009)]. The most highly represented motif corresponds to the canonical GBS. (B) Canonical GBS reported with the strength of Gcm binding and references. (C) Distance between the DamID peaks and the closest canonical GBS. (D) Distribution of the number of canonical GBSs per kilobase in the whole genome and under the DamID peaks. The box delimits the second and third quartiles; the thick black bar indicates the median for the two populations; the *P*-values are indicated as follows: ns = nonsignificant (*P* > 0.05); *P = 0.05–0.01; **P = 0.01–0.001; ***P < 0.001. (E) Distribution of conservation scores of canonical GBSs in the whole genome and under the DamID peaks. Box, thick black bar; asterisk, as in D. (F) Coding status of the genomic region covered by the DamID.

The *Drosophila* genome (version BDGP R5/Dm3) was scanned for the canonical Gcm binding sites (GBSs) listed in Figure 1B. For each GBS, the conservation score, which was calculated from 12 *Drosophila* species, mosquito, honeybee, and red flour beetle (Blanchette *et al.* 2004; Siepel *et al.* 2005), was taken from the Conservation track (multiz15way) on the University of California Santa Cruz (UCSC) Genome Browser. The GBSs located within 1 kb of DamID peaks were compared with the whole population of GBSs (Figure 1E). An *F*-test was used to compare the variance of the two populations, and a *t*-test for unequal sample variance was used to calculate the *P*-value.

### Comparison with expression profiling data

**Figure 2:**
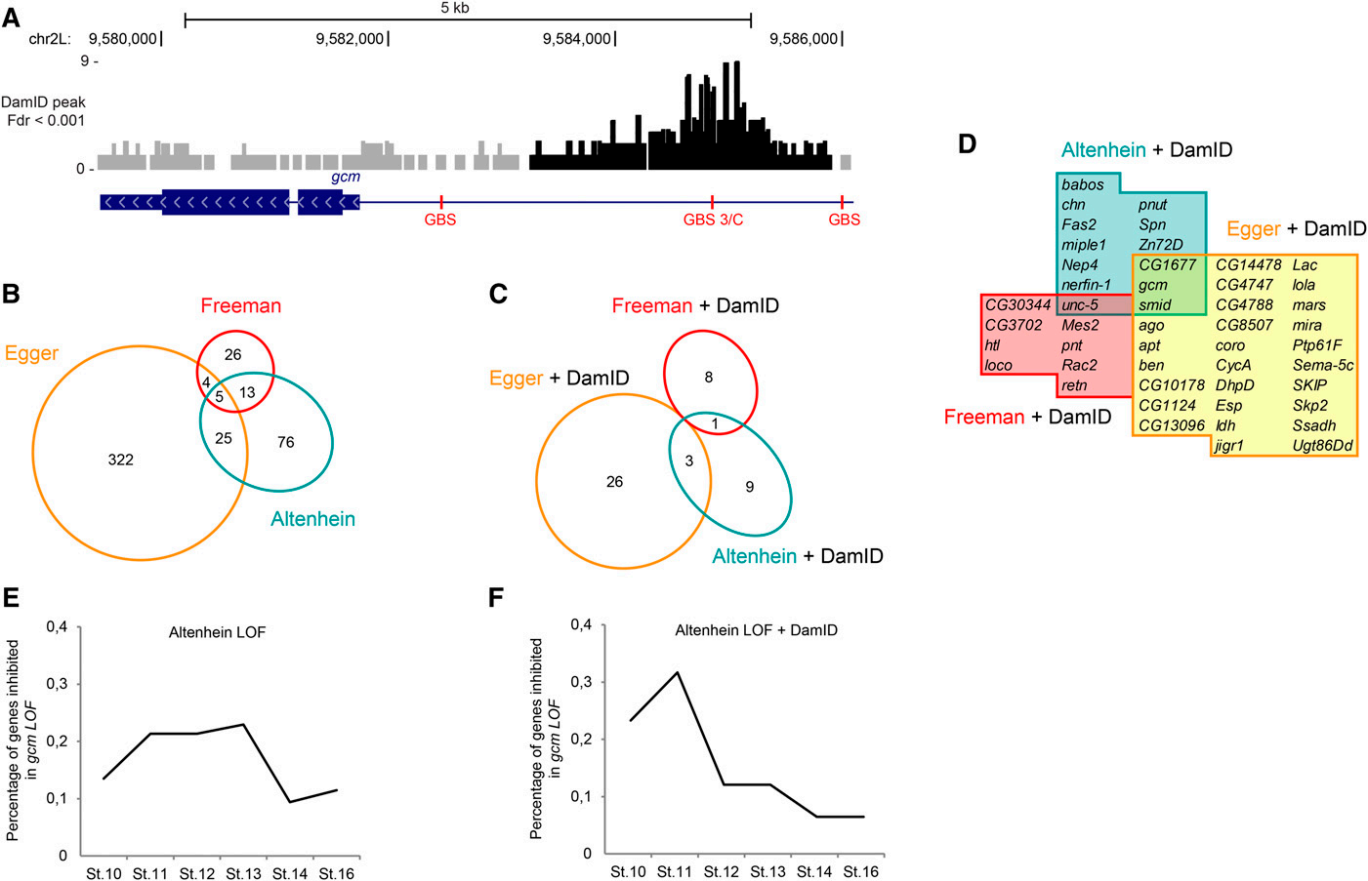
Known targets of Gcm are found in the DamID screen. (A) Schematic representation of the *gcm* locus. The gene is indicated by the blue rectangles, thin ones indicating the untranslated regions (UTR) and thick ones indicating the coding exons (CDSs); pale blue arrowheads indicate the direction of transcription. In this and the following figures, GBSs are indicated in red, and the histograms above the locus show a region of 1 kb on each side of a DamID peak scoring a FDR < 0.001. The histograms in gray indicate the nonsignificant DamID peaks with a FDR > 0.001. The genomic coordinates of the loci (genome version BDGP R5/Dm3) are indicated above the histograms. (B) Euler diagram representing the overlap between the downstream targets of Gcm identified by Altenhein *et al.* (2002), Freeman *et al.* (2003), and Egger *et al.* (2002). The size of each area is proportional to the number of genes included in the category. (C) Subset of genes identified in the three transcriptome assays mentioned in B that are also identified as direct targets by the DamID screen. The size of each area is proportional to the number of genes included in the category. (D) Names of the genes in common between the screens of Altenhein *et al.* (2002), Freeman *et al.* (2003), and Egger *et al.* (2003) and the DamID. (E) Distribution of the genes whose expression decreases in *gcm* LOF according to the earliest developmental stages at which they were identified [data set Altenhein *et al.* (2002)]. (F) Same distribution as in E but for genes present in both the DamID screen and the Altenhein *et al.* (2002) LOF data set.

The data set from Freeman *et al.* (2003) was retrieved directly from the publication. For Egger’s data set, the raw data were retrieved and analyzed as described in the paper (Egger *et al.* 2002) (intensities >50 and fold change >1.5) with a more restrictive *P*-value (<0.001). The data set from Altenhein *et al.* (2006) comprising the filtered and tested genes for the *gcm* gain of function (GOF) and *gcm* loss of function (LOF) was retrieved, and all genes giving nonspecific or negative *in situ* hybridizations (ISHs) were removed to make the Venn diagrams in Figure 2. The R package VennDiagram was used to draw the diagrams in Figure 2, B and C (Chen and Boutros 2011). The gene names for all data sets then were converted to FlyBase gene numbers (Fbgn) for comparison with the DamID genes using the FlyBase conversion tool (dos Santos *et al.* 2015).

The expression profiles of the DamID genes were compared to the expression profile of *gcm* in embryos using the *in situ* data produced by the Berkeley Drosophila Genome Project on embryos (Tomancak *et al.* 2002, 2007; Hammonds *et al.* 2013) (Table 1, Table 2, and Supporting Information, Table S1, column O).

**Table 1:** Targets of Gcm involved in nervous system development

Gene symbol	Annotation	References
*beat-Ia,^a^ beat-Ib,^a^ beat*-IIIb,*^b^ beat-IIIc*,*^b^ beat-VI*, *beat-VII*, *btl*, *Dscam*,*^b^ Dscam3,^a^ Dscam4*, *fas*, *Lar*, *Ptp99A*, *robo*, *robo2*, *robo3*,*^b^ Trim9*,*^b^ tutl^b^*	Axon guidance	Seeger *et al.* (1993); Desai *et al.* (1996); Krueger *et al.* (1996); Lekven *et al.* (1998); Rajagopalan *et al.* (2000); Pipes *et al.* (2001); Sun *et al.* (2001); Jhaveri *et al.* (2004); Hiramoto and Hiromi (2006); Al-Anzi and Wyman (2009); Hofmeyer and Treisman (2009); Prakash *et al.* (2009); Zeev-Ben-Mordehai *et al.* (2009); Song *et al.* (2011); Armitage *et al.* (2012); Kim *et al.* (2013)
*btl*, *fra*,*^b^ sty*, *ths*, *unc-5*	Axon ensheatment and glial cell migration	Klambt *et al.* (1992); Shishido *et al.* (1997); Franzdottir *et al.* (2009); von Hilchen *et al.* (2010); Mukherjee *et al.* (2012)
*Atp-α*, *bib*,*^b^ CG13248*,*^b^ CG13384*, *CG30344*,*^b^ Fas2*, *Fas3*,*^b^ Lac*,*^b^ Sln*, *VGAT*, *wun^a^*	Blood-brain barrier (amino acid transport, septate junction)	Karlstrom *et al.* (1993); Strigini *et al.* (2006); DeSalvo *et al.* (2014); Limmer *et al.* (2014); Deligiannaki *et al.* (2015)
*Htl^b^*	Blood-brain barrier, axon ensheatment, and glial cell migration	Shishido *et al.* (1993); Shishido *et al.* (1997); DeSalvo *et al.* (2014)
*Babos^b^*	Dendritic plasticity	Yuan *et al.* (2011)
*Jbug^b^*	Epileptic seizure	Goldstein and Gunawardena (2000); Song and Tanouye (2006)
*CG13506*,*^a^* ImpL2,*^b^ InR*	Insulin regulation	Garbe *et al.* (1993); Claeys *et al.* (2002); Song *et al.* (2003); Yuan *et al.* (2011); Sarraf-Zadeh *et al.* (2013)
*gcm*,*^b^ gcm2*,*^b^ hkb*,*^b^ loco*,*^b^ pnt*,*^b^ retn^b^*	Glial cell development	Granderath *et al.* (1999); Kammerer and Giangrande (2001); Shandala *et al.* (2003); Yuasa *et al.* (2003); De Iaco *et al.* (2006); Popkova *et al.* (2012)
*Dr*,*^b^ vnd^b^*	Late embryonic brain development	Sprecher and Hirth (2006)
*CycE*,*^b^ Dl*,*^b^ drk*,*^b^ Egfr*,*^b^ N*,*^a^ Ras85D^a^*	Longitudinal glia precursor division	Hidalgo *et al.* (2001); Griffiths and Hidalgo (2004); Berger *et al.* (2005a,b); Thomas and van Meyel (2007)
*brat*,*^b^ E(spl)m5*,*^b^ E(spl)m7*,*^b^ E(spl)m8*,*^b^ E(spl)mβ*,*^b^ E(spl)mδ*,*^b^ E(spl)mɣ*,*^a^ lola*,*^b^ mira*,*^b^ pros^b^*	Neural stem cell regulation	Neumuller *et al.* (2011); Carney *et al.* (2012); Zacharioudaki *et al.* (2012)
*ase*,*^b^ ato*,*^b^ ci*,*^b^ d*,*^b^ dally*, *ds*, *ft*,*^b^ hbs*, *hh*, *l(1)sc*,*^b^ Pka-C1*,*^a^ ptc*, *rdx*,*^b^ rho*,*^b^ Ser*, *th*,*^b^ tll^b^*	Optic lobe development	Gonzalez *et al.* (1989); Huang and Kunes (1996); Rudolph *et al.* (1997); Huang and Kunes (1998); Daniel *et al.* (1999); Chotard *et al.* (2005); Sugie *et al.* (2010); Yasugi *et al.* (2010); Kawamori *et al.* (2011); Perez-Gomez *et al.* (2013); Onel *et al.* (2014)
*DIP-β*,*^b^ DIP-θ*,*^α^ dpr11*,*^α^ dpr15*,*^α^ dpr2*, *dpr3*, *dpr4*, dpr5,*^b^ dpr8*	Enriched in glia	DeSalvo *et al.* (2014)
*CG34371*, *CG42313*, *CG42389*, *E(spl)m4*, *E(spl)m6*, *kek6*, *wake^b^*	Expressed in the CNS	Bailey and Posakony (1995); Graveley *et al.* (2011)

a Genes not coexpressed in embryos according to the Berkeley Drosophila Genome Project *in situ* database. No mark for genes that were not assayed.

b Genes coexpressed in embryos with *gcm*. No mark for genes that were not assayed.

**Table 2:** Targets of Gcm involved in immune system development

Gene symbol	Annotation	References
*att-ORFA*, *CanA1*,*^a^ coro*,*^b^ Crag*,*^a^ dnr1*,*^b^ dos*,*^a^ E2f1*,*^b^ jumu*,*^b^ loco*,*^b^ lola*,*^b^ mtd*,*^b^ nub*,*^b^ os*, *par-1*,*^a^ Pli*, *scny*,*^a^ shg*,*^b^ Stat92E*,*^b^ tefu*, *wun*,*^a^ zfh1^b^*	Antimicrobial humoral response (response to fungi, response to Gram-negative bacteria)	Kleino *et al.* (2005); Dijkers and O’Farrell (2007); Kim *et al.* (2007); Jin *et al.* (2008); Avadhanula *et al.* (2009); Cronin *et al.* (2009); Guntermann *et al.* (2009); Petersen *et al.* (2012); Wang *et al.* (2012); Dantoft *et al.* (2013); Engel *et al.* (2014); Ji *et al.* (2014)
*Atg18*,*^b^ Atg5*,*^a^ Atg8b*,*^a^ Atg9*,*^a^ hid*,*^b^ Rab7^a^*	Autophagy	Gorski *et al.* (2003); Hou *et al.* (2008); Yano *et al.* (2008); Ren *et al.* (2009)
*CG11313^a^*	Coagulation	Karlsson *et al.* (2004)
*if*, *sn^a^*	Hemocyte migration	Zanet *et al.* (2)009; Siekhaus *et al.* (2010)
*alpha-Man-IIb*, *Gl*,*^a^ Rac2*,*^b^ RhoGEF3*,*^b^ Zir^a^*	Melanotic encapsulation of foreign targets	Howell *et al.* (2012); Mortimer *et al.* (2012)
*crq*,*^b^ dally*, *DMAP1*,*^a^ Dscam*,*^b^ pnt*,*^b^ Traf4^b^*	Phagocytosis	Franc *et al.* (1999); Watson *et al.* (2005); Stroschein-Stevenson *et al.* (2006); Avet-Rochex *et al.* (2007); Zhu and Zhang (2013)
*fz*, *fz2*,*^b^ wg*,*^b^ wntD^b^*	Wnt mediated inflammatory cascade	Zhang and Carthew (1998); Gordon *et al.* (2005); Sen and Ghosht (2008)
*Alk*, *aop*,*^a^ Egfr*,*^b^ N*,*^a^ Ras85D^a^*	Hemocytes proliferation	Lebestky *et al.* (2003); Zettervall *et al.* (2004)
*Antp*,*^b^ Gale*,*^b^ lolal*,*^b^ mRpL53*, *pnr*,*^b^ Ser*, *tup^b^*	Lymph gland development	Lebestky *et al.* (2003); Mandal *et al.* (2004); Han and Olson (2005); Mandal *et al.* (2007); Tao *et al.* (2007); Mondal *et al.* (2014)
*dpp*, *gcm*,*^b^ gcm2*,*^b^ htl*,*^b^ l(3)mbn*,*^a^ Pvf3*,*^b^ ush^b^*	Plasmatocytes differentiation	Konrad *et al.* (1994); Bernardoni *et al.* (1997); Lebestky *et al.* (2000); Fossett *et al.* (2001); Kammerer and Giangrande (2001); Alfonso and Jones (2002); Cho *et al.* (2002); Muratoglu *et al.* (2006); Frandsen *et al.* (2008); Parsons and Foley (2013)
*apt*,*^b^ Pen*, *Ptp61F*,*^b^ Socs36E*,*^b^ Socs44A^a^*	Repression of lamellocyte differentiation	Kussel and Frasch (1995); Callus and Mathey-Prevot (2002); Rawlings *et al.* (2004); Baeg *et al.* (2005); Muller *et al.* (2005); Sorrentino *et al.* (2007); Starz-Gaiano *et al.* (2008); Stec *et al.* (2013)

a Genes not coexpressed in embryos according to the Berkeley Drosophila Genome Project *in situ* database. No mark for genes that were not assayed.

b Genes coexpressed in embryos with *gcm*. No mark for genes that were not assayed.

### Fly strains and immunolabeling

Flies were raised on standard medium at 25°. The following strains were used: *gcmGal4*, *UASmCD8GFP*/*CyO*, *Tb1* (*gcmGal4*, *UASGFP* in the text) (Soustelle and Giangrande 2007), *y1v1*; *P(TRiP.JF01075)attP2* (*UASgcmRNAi* in the text) (Bloomington B#31519), *repoGal80* (gift of B. Altenhein), and *enGal4* (Bloomington B#30564) weres crossed with *Oregon R* flies to generate *enGal4*/*+* or with *UASgcm F18A* flies (Bernardoni *et al.* 1998) to generate *enGal4*/*+*; *UASgcm*/*+* flies (Figure 4), and *gcmGal4*, *UASmCD8GFP* was recombined with *repoGal80* to produce the line *gcmGal4*, *UASmCD8GFP*, *repoGal80/CyO*. Overnight lays of *Drosophila* embryos were used for Figure 4. In Figure 5, *Drosophila* central nervous systems (CNSs) were dissected and labeled as described previously (Ceron *et al.* 2001). The primary antibodies used were rat anti-Ci [1:100; supernatant from the Developmental Studies Hybridoma Bank (DSHB)], mouse anti-Ptc (1:100; supernatant from the DSHB), mouse anti-Smo (1:100; supernatant from the DSHB), rat anti-Elav (1:200; supernatant from the DSHB), chicken anti-GFP (1:1000; Abcam #13970), and rabbit anti-Dh31 [1:500; kindly provided by J. Veenstra (Veenstra *et al.* 2008; Veenstra 2009)]. Secondary antibodies conjugated with FITC, Cy3, or Cy5 (Jackson) were used at 1:500. DAPI was used at 100 ng/ml for nuclear counterstaining. Embryos and brains were mounted in VECTASHIELD (Vector) mounting medium and analyzed by confocal microscopy (Leica SP5) using identical settings between controls and mutants (*gcm* GOF and hypomorph).

### Gene Ontology (GO) term and protein domain enrichment analysis

The GO term and protein domain enrichment analyses were performed using the DAVID functional classification tool (Huang *et al.* 2009a,b).

### Luciferase assay in S2 cells

**Figure 3:**
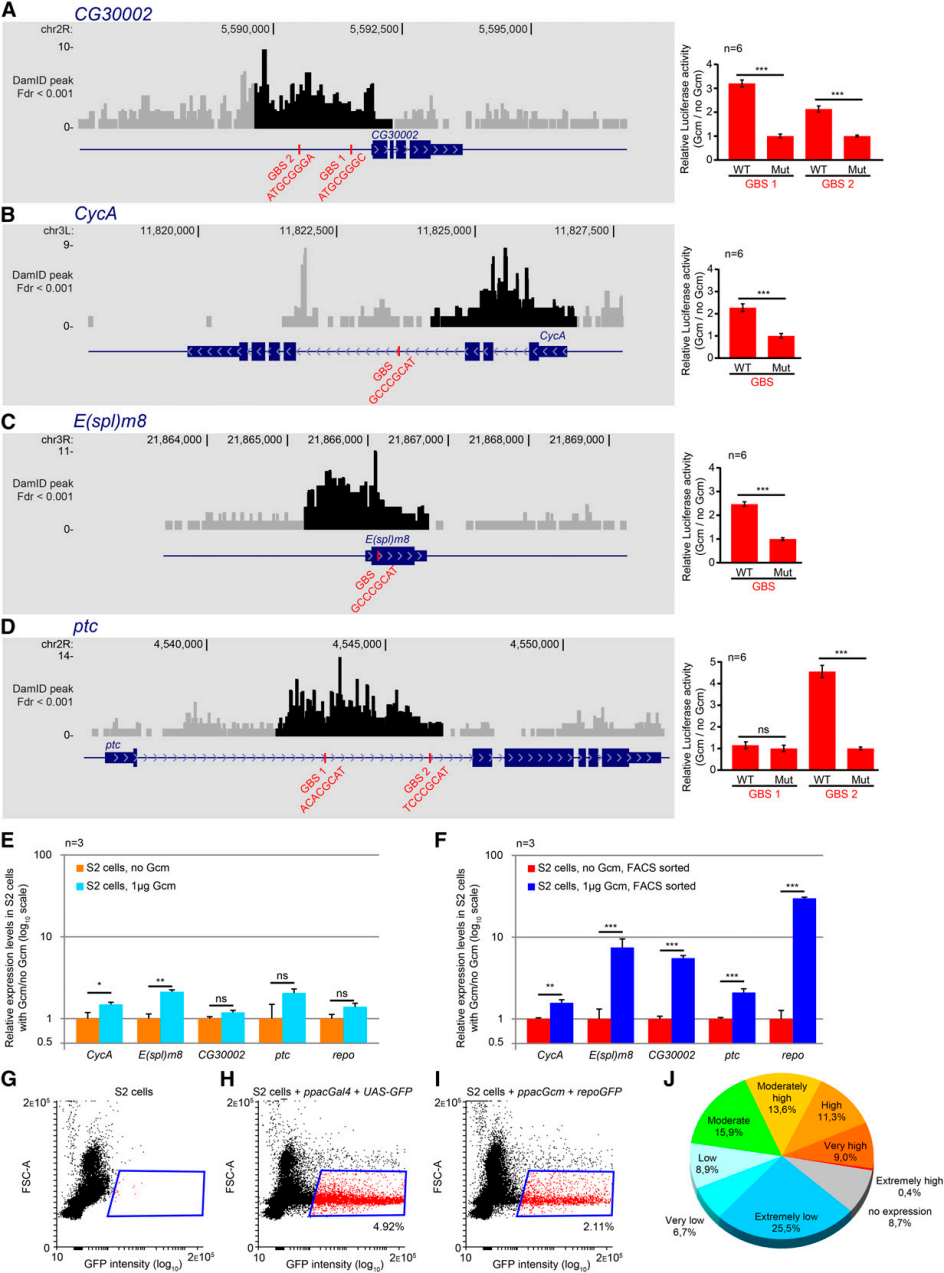
Validation of new Gcm targets identified by the DamID screen. (A–D) The left panels represent the loci containing the DamID peaks and the GBSs for *CG30002* (A), *CycA* (B), *E(spl)m8* (C), and *ptc* (D) (as described in Figure 2A), and the histograms on the right (in red) represent the results of the luciferase assays carried out on each GBS. The red bars indicate enrichment of luciferase activity in the presence of Gcm compared to no transfected Gcm (“no Gcm”); bars indicate SEM; and *n* represents the number of independent transfection assays. *p*-values are as indicated as Figure 1D. (E) Histogram representing the endogenous levels of expression of *CycA*, *E(spl)m8*, *CG30002*, *ptc*, and *repo* in S2 cells with and without transfected Gcm. The *y*-axis is in log_10_ scale; error bars and *P*-values are calculated as indicated in Figure 3, A–D. (F) Same as E after FACS sorting of the Gcm^+^ cells. (G–I) FACS analyses of S2 cells (G), of S2 cells transfected with the Gal4 expression vector *ppacGal4* and the GFP reporter *UAS-GFP* (H), and of S2 cells transfected with *ppacGcm* and *repoGFP* (I). The dotplots show the forward scatter area (FSC-A) on the *y*-axis and the GFP intensity on the *x*-axis. The area in blue indicates the GFP^+^ cells that were sorted for further analysis, and the number under the area indicates the percentage of cells that are GFP^+^. (J) Diagram representing the distribution of the DamID targets according to their levels of expression in stage 10–11 embryo (4- to 8-hr embryo in modENCODE development RNA-seq).

For *CG30002*, *CycA*, *E(spl)m8*, and *ptc* (Figure 3, A–D), sense and antisense oligonucleotides covering the GBSs in each gene were synthesized with flanking restriction sites for *Kpn*I at the 5′ extremity and for *Nhe*I at the 3′ extremity. Each pair of oligonucleotides was designed with the wild-type (WT) GBS and a mutated GBS that is not bound by Gcm (mutated for nucleotides 2, 3, 6, and/or 7. In Table 3, the GBS and restriction sites are indicated by capital letters. For each GBS, the WT and mutant double-stranded probes were prepared as follows: 1 nmol of forward probe and 1 nmol of reverse probe were combined in 10 mM Tris, pH 7.5, 1 mM EDTA, and 50 mM NaCl in 100 μl of total solution. The mix was incubated for 1 min at 95° in a heating block, and then the heating block was turned off and allowed to cool to 25°. Then 2 μg of annealed oligonucleotides was digested with 20 units of *Kpn*I [New England Biolabs (NEB) #R3142S] and 20 units of *Nhe*I (NEB #R3131S) in CutSmart buffer (NEB #B7204S) for 90 min at 37°. The digested double-stranded probes then were cleaned using a PCR Clean-Up Kit [Macherey-Nagel (MN) #740609] according to the manufacturer’s instructions.

**Table 3:** Oligonucleotides used to generate the *pGL4.23* vectors used in S2 cells

Probe name	Probe sequence
CG30002_GBS1_mutF	gagaGGTACCtttgccgaaaaatgttcgggtAGTCGTTGcatacaatatccgctgaaacgGCTAGCgaga
CG30002_GBS1_mutR	tctcGCTAGCcgtttcagcggatattgtatgCAACGACTacccgaacatttttcggcaaaGGTACCtctc
CG30002_GBS1_wtF	gagaGGTACCtttgccgaaaaatgttcgggtATGCGGGCcatacaatatccgctgaaacgGCTAGCgaga
CG30002_GBS1_wtR	tctcGCTAGCcgtttcagcggatattgtatgGCCCGCATacccgaacatttttcggcaaaGGTACCtctc
CG30002_GBS2_mutF	gagaGGTACCtgtttgtgggcttgttctgaaaAGTCGTTGcagtgggttatgagaacaaaGCTAGCgaga
CG30002_GBS2_mutR	tctcGCTAGCtttgttctcataacccactgCAACGACTtttcagaacaagcccacaaacaGGTACCtctc
CG30002_GBS2_wtF	gagaGGTACCtgtttgtgggcttgttctgaaaATGCGGGAcagtgggttatgagaacaaaGCTAGCgaga
CG30002_GBS2_wtR	tctcGCTAGCtttgttctcataacccactgTCCCGCATtttcagaacaagcccacaaacaGGTACCtctc
cycA_GBS_mutF	gagaGGTACCctccacggccaacttggaattcCAACGACTcagctcatcgaattccgcctGCTAGCgaga
cycA_GBS_mutR	tctcGCTAGCaggcggaattcgatgagctgAGTCGTTGgaattccaagttggccgtggagGGTACCtctc
cycA_GBS_wtF	gagaGGTACCctccacggccaacttggaattcGCCCGCATcagctcatcgaattccgcctGCTAGCgaga
cycA_GBS_wtR	tctcGCTAGCaggcggaattcgatgagctgATGCGGGCgaattccaagttggccgtggagGGTACCtctc
E(spl)m8_GBS_mutF	gagaGGTACCatgctggagcgccagcgacgtCAACGACTgaacaagtgcctggacaacctGCTAGCgaga
E(spl)m8_GBS_mutR	tctcGCTAGCaggttgtccaggcacttgttcAGTCGTTGacgtcgctggcgctccagcatGGTACCtctc
E(spl)m8_GBS_wtF	gagaGGTACCatgctggagcgccagcgacgtGCCCGCATgaacaagtgcctggacaacctGCTAGCgaga
E(spl)m8_GBS_wtR	tctcGCTAGCaggttgtccaggcacttgttcATGCGGGCacgtcgctggcgctccagcatGGTACCtctc
ptc_GBS1_mutF	gagaGGTACCcatacacacacacacacacacCAACGACTcaacacacacacacacacgaaGCTAGCgaga
ptc_GBS1_mutR	tctcGCTAGCttcgtgtgtgtgtgtgtgttgAGTCGTTGgtgtgtgtgtgtgtgtgtatgGGTACCtctc
ptc_GBS1_wtF	gagaGGTACCcatacacacacacacacacacACACGCATcaacacacacacacacacgaaGCTAGCgaga
ptc_GBS1_wtR	tctcGCTAGCttcgtgtgtgtgtgtgtgttgATGCGTGTgtgtgtgtgtgtgtgtgtatgGGTACCtctc
ptc_GBS2_mutF	gagaGGTACCagcaggaagtgcaggatgctaCAACGACTaagtatgagtatcttccccatGCTAGCgaga
ptc_GBS2_mutR	tctcGCTAGCatggggaagatactcatacttAGTCGTTGtagcatcctgcacttcctgctGGTACCtctc
ptc_GBS2_wtF	gagaGGTACCagcaggaagtgcaggatgctaTCCCGCATaagtatgagtatcttccccatGCTAGCgaga
ptc_GBS2_wtR	tctcGCTAGCatggggaagatactcatacttATGCGGGAtagcatcctgcacttcctgctGGTACCtctc

Then 1 μg of luciferase reporter plasmid *pGL4.23[luc2*/*minP]* (*pGL4.23*) (Promega #E841A) was digested with *Kpn*I and *Nhe*I as described previously; after 90 min at 37°, 20 units of alkaline phosphatase, calf intestinal (CIP; Promega #M0290S) was added to the plasmid and incubated for 1 hr at 37°. The plasmid then was cleaned using a PCR Clean-Up Kit (MN #740609).

Then 50 ng of digested luciferase plasmid was combined with the digested annealed probes (ratio plasmid/probe = 1:6), 400 units of ligase (NEB #M0202S), and ligation buffer (NEB #B0202S) and incubated overnight at 18°. The ligated plasmids then were dialyzed for 30 min on membrane filters (Millipore #VSWP02500) and amplified using the Plasmid DNA Purification Kit (MN #740410) according to the manufacturer’s instructions.

Transfections of *Drosophila* S2 cells were carried out in 12-well plates using Effectene transfection reagent (Qiagen #301427) according to the manufacturer’s instructions. Cells were transfected with 0.5 μg *pPac-lacZ*, 0.5 μg *pGL4.23* carrying the indicated GBS, 0.5 μg *pPac-gcm* (Miller *et al.* 1998) or 0.5 μg *pPac* (Krasnow *et al.* 1989). Then, 48 hr after transfection, cells were collected, washed once in cold PBS, and resuspended in 100 μl of lysis buffer (25 mM Tris-phosphate, pH 7.8, 2 mM EDTA, 1 mM DTT, 10% glycerol, and 1% Triton X-100). The suspensions were frozen and thawed four times in liquid nitrogen and centrifuged for 30 min at 4° at 13,000 × *g*. The luciferase and LacZ activities were measured in triplicate for each sample. For LacZ measurements, 20 μl of lysate was mixed with 50 μl of β-galactosidase assay buffer (60 mM Na_2_PO_4_, 40 mM NaH_2_PO_4_, 10 mM KCl, 1 mM MgCl_2_, and 50 mM β-mercaptoethanol) and 20 μl of *ortho*-nitrophenyl-β-galactoside (ONPG, 4 mg/ml) and incubated at 37° for 20 min. The reaction was stopped by adding 50 μl of 1 M Na_2_CO_3_, and the optical density at 415 nm was measured. For luciferase activity, 10 μl of protein lysate was analyzed on an opaque 96-well plate (Packard Instruments #6005290) with a Berthold Microluminat LB96P Luminometer by injecting 50 μl of luciferase buffer (20 mM Tris-phosphate, pH 7.8, 1 mM MgCl_2_, 2.5 mM MgSO_4_, 0.1 mM EDTA, 0.5 mM ATP, 0.5 mM luciferine, 0.3 mM coenzyme A, and 30 mM DTT). For both LacZ and luciferase assays, background levels were estimated using lysate from nontransfected S2 cells. The relative luciferase levels were calculated as follows: first, the background was subtracted from each value, and then the average values of the technical triplicate were calculated. From there, the luciferase activity of each sample was normalized to the LacZ activity (luciferase activity/LacZ activity) to correct for transfection efficiency variability, and the ratio luciferase with Gcm/luciferase without Gcm was calculated. For each WT and mutant GBS, biological triplicates were carried out.

### S2 cell FACS and quantitative PCR (qPCR)

S2 cells were plated in six-well plates, 6 million cells per well, in 1.5 ml of Schneider medium complemented with 10% fetal calf serum (FCS) and 0.5% penicillin and 0.5% streptomycin (PS). Cells were transfected 12 hr after plating using Effectene Transfection Reagent (Qiagen). Briefly, 2 µg of *pPac-gal4* vector and 1 µg of *pUAS-GFP* for the negative control and 2 µg of *pPac-gcm* (Miller *et al.* 1998) and 1 µg of *4.3kb repo-GFP* (*repoGFP*) (Laneve *et al.* 2013) for the *gcm* GOF were mixed with 90 µl of EC buffer and 24 µl of enhancer and incubated for 5 min at room temperature, and then 25 µl of Effectene was added, and the mix was incubated at room temperature for 20 min. Then 600 µl of Schneider medium + 10% FCS + 0.5% PS was added to the mix before spreading it on the cells. At 48 hr after transfection, cells were sorted on a BD FACSAria according to GFP expression to obtain more than 80% of transfected cells in the sample. The RNA then was extracted using TRI Reagent (Sigma), and 1 µg of RNA per sample was DNAse treated with RNAse-free DNAse 1 (Thermo Fisher) and reverse transcribed with Superscript II (Invitrogen). qPCR was performed on a LightCycler 480 (Roche) with SYBR master (Roche) on the equivalent of 5 ng of reverse-transcribed RNA with the primer pairs listed in Table S3. Each PCR was carried out in triplicate on at least three biological replicates. The quantity of each transcript was normalized to the quantity of the housekeeping genes *Glyceraldehyde 3 phosphate dehydrogenase 1* (*Gapdh1*) and *Actin 5c* (*Act5c*). The *P*-values were measured comparing the control with the transfected cells using Student’s *t*-test (bars = SEM).

### qPCR on *gcm*-overexpressing embryos

RNA extraction was carried out on 50 to 100 *enGal4*/*+* or *enGal4*/*+*; *UASgcm*/*+* stage 13–14 embryos (at 25°, 2 hr of egg laying, 9 hr and 20 min of incubation before collection) using TRI Reagent (Sigma). RNA extraction and qPCR were performed as described for the S2 cells in triplicate.

### Conversion to human orthologs

The Drosophila RNAi Screening Center (DRSC) integrative ortholog prediction tool (Hu *et al.* 2011) was used to retrieve the human orthologs of all Gcm targets identified in *Drosophila* by the DamID screen. All human genes with a weighted score > 1 were selected.

### qPCR in HeLa cells

HeLa cells were plated in six-well plates, 400,000 cells per well, in 1.6 ml of Dulbecco’s Modification of Eagle’s Medium (DMEM) complemented with 5% FCS and gentamycin. Cells were transfected 12 hr after plating using Effectene Transfection Reagent (Qiagen). Briefly, 1 µg of *pCIG* vector, 1 µg of *pCIG* vector expressing mouse *Gcm1* (*pCIG-mGcm1*) (Soustelle *et al.* 2007), or 1 µg of *pCIG* vector expressing mouse *Gcm2* (*pCIG-mGcm2*) were mixed with 100 µl of EC buffer and 8 µl of enhancer and incubated for 5 min at room temperature, and then 10 µl of Effectene was added, and the mix was incubated at room temperature for 20 min. Then 200 µl of DMEM + 5% FCS + gentamycin was added to the mix before spreading it on the cells, and 48 hr after transfection, the RNA was extracted using TRI Reagent (Sigma). Then 1 µg of RNA per sample was DNAse treated with RNAse-free DNAse 1 (Thermo Fisher) and reverse transcribed with Superscript II (Invitrogen). qPCR was performed on a LightCycler 480 (Roche) with SYBR master (Roche) on the equivalent of 5 ng of reverse-transcribed RNA with the primer pairs listed in Table S3. Each PCR was carried out in triplicate on at least three biological replicates. The quantity of each transcript was normalized to the quantity of the housekeeping genes *Glyceraldehyde 3 phosphate dehydrogenase* (*GAPDH*) and *Actin Beta* (*ACTB*). The *P*-values were measured comparing the control with the transfected cells using Student’s *t*-test (bars = SEM).

### Luciferase assay in HeLa cells

For *GCM1* and *GCM2* (Figure 9), oligonucleotides surrounding the GBSs were designed with flanking restriction sites for *Kpn*I at the 5′ extremity and for *Nhe*I at the 3′ extremity (in Table 4, the GBS and restriction sites are indicated by lowercase letters). Each pair of oligonucleotides was used to amplify the genomic region encompassing the GBSs on HeLa genomic DNA using Expand High Fidelity System DNA polymerase (Roche). The amplicons were digested with 20 units of *Kpn*I (NEB #R3142S) and 20 units of *Nhe*I (NEB #R3131S) in CutSmart buffer (NEB #B7204S) for 90 min at 37°. The digested amplicons then were cleaned using a PCR Clean-Up Kit (MN #740609) according to the manufacturer’s instructions. Then 1 μg of luciferase reporter plasmid *pGL4.23[luc2*/*minP]* (*pGL4.23*) (Promega #E841A) was digested with *Kpn*I and *Nhe*I as described previously. After 90 min at 37°, 20 units of CIP (Promega #M0290S) was added to the plasmid and incubated for 1 hr at 37°. The plasmid then was cleaned using a PCR Clean-Up Kit (MN #740609) according to the manufacturer’s instructions, and 50 ng of digested luciferase plasmid was combined with the digested amplicons (ratio of plasmid/probe = 1:6), 400 units of ligase (NEB #M0202S), and ligation buffer (NEB #B0202S) and incubated overnight at 18°. The ligated plasmids then were dialyzed for 30 min on membrane filters (Millipore #VSWP02500) and amplified using the Plasmid DNA Purification Kit (MN #740410) according to the manufacturer’s instructions. These plasmids were used for the luciferase assay (Figure 9) and as templates for mutagenesis. To mutagenize the reporters, primers overlapping the GBSs were designed with mutations for nucleotides at position 2, 3, 6, and/or 7 in the GBSs (in Table 4, the GBS and restriction sites are indicated by lowercase letters).

**Table 4:** Oligonucleotides used to generate the *pGL4.23* vectors used in HeLa cells

Probe name	Probe sequence
GCM1 F	GAGAggtaccCAGAGCCTGCTGGGACTTGA
GCM1 R	TCTCgctagcTAGCTGGGATTACAGGCACG
GCM1 GBS1mut forward	GAGAggtaccCAGAGCCTGCTGGGACTTGAaaaagacgTAAGATTTTCACGACACAGTGCTGT
GCM1 GBS2mut reverse	GAACAGTAACGATATTGTCTcgtattttGCAAATTTTGTTATAACTAATTGGA
GCM1 GBS2mut forward	TTAGTTATAACAAAATTTGCaaaatacgAGACAATATCGTTACTGTTCAGGGT
GCM1 GBS3mut reverse	TCTCgctagcTAGCTGGGATTACAGGCACGccacaaaaCACCCAGCTAATTTTTGTATTTTCA
GCM2 F	GAGAggtaccCAGGTAAGTGAACCGGGTGT
GCM2 R	TCTCgctagcGGTAGAGACGGGGTTTCTCC
GCM2 GBS1mut forward	GAGAggtaccCAGGTAAGTGAACCGGGTGTggtcgttcACGCGGGGCGCTGTCCATCCGAAGG
GCM2 GBS2mut reverse	GCCTCAGAAACCCAGAAATTtgtcgtttATGTGTGTGTGTGTGTGTGTGTGTG
GCM2 GBS2mut forward	ACACACACACACACACACATaaacgacaAATTTCTGGGTTTCTGAGGCCCTCT
GCM2 GBS3mut reverse	TCTCgctagcGGTAGAGACGGGGTTTCTCCagttttttCAGGCTGGTCTTGAACTCCCGACCT

For each gene, PCR was performed using 5 ng of *pGL4.23* containing the WT locus with Expand High Fidelity System DNA polymerase (Roche). A first round of PCRs was carried out to generate the amplicon containing the first and second mutated GBSs and the amplicon containing the second and third mutated GBSs with the following primer pairs: GBS1mut forward/GBS2mut reverse and GBS2mut forward/GBS3mut reverse. The two amplicons then were combined using the primers GBS1mut forward and GBS3mut reverse and inserted into *pGL4.23*.

HeLa cells were plated in 24-well plates, 60,000 cells per well, in 350 µl of DMEM complemented with 5% FCS and gentamycin. Cells were transfected 12 hr after plating using Effectene Transfection Reagent (Qiagen). Briefly, 2.5 ng of *pGL4.75* vector, 250 ng of *pCIG* vector expressing either mouse *Gcm1* (*pCIG-mGcm1*) (Soustelle *et al.* 2007) or mouse *Gcm2* (*pCIG-mGcm2*) or empty, and 250 ng of *pGL4.23* vector containing the GBS WT or mutant were mixed with 60 µl of EC buffer and 4 µl of enhancer and incubated 5 min at room temperature; then 5 µl of Effectene was added, and the mix was incubated at room temperature for 20 min. Then 100 µl of DMEM + 5% FCS + gentamycin was added to the mix before spreading it on the cells, and 48 hr after transfection, the luciferase assay was performed using the Dual-Luciferase Reporter Assay System (Promega) according to the manufacturer’s instructions with a Berthold Microluminat LB96P Luminometer.

### Data availability

All data are joined with the publication in Supporting Information.

## Results

### The DamID screen identifies loci containing GBSs

To identify the genes directly regulated by Gcm, we mapped its binding sites using a genome-wide DamID screen (van Steensel and Henikoff 2000; van Steensel *et al.* 2001). Briefly, the *Escherichia coli* DNA adenine methyltransferase (Dam) was fused N-terminal to the full-length Gcm coding sequences. Thus, wherever Gcm binds, the Dam methylates the surrounding DNA. The methylated DNA then can be identified by microarray. In our case, the Dam-Gcm screen was performed on *Drosophila* embryos at stage 11, when Gcm expression peaks. Because Gcm is expressed in several cell types: glia, hemocytes, and tendon cells (Soustelle *et al.* 2004), as well as neuronal (Chotard *et al.* 2005; Soustelle and Giangrande 2007) and peritracheal cell subsets (Laneve *et al.* 2013), we decided to search for all its direct targets and did not restrict expression of the Dam-Gcm fusion to a specific cell type.

Overall, 4863 DamID peaks were identified. Motif enrichment analysis using the MICRA tool (Southall and Brand 2009) revealed enrichment for the motif ATGCGGG at the loci bound by the Dam-Gcm fusion (Figure 1A). This motif is closely related to most of the GBSs previously described and validated functionally (Figure 1B) (Akiyama *et al.* 1996; Schreiber *et al.* 1997; Miller *et al.* 1998; Ragone *et al.* 2003). Up to 83% of the loci identified in the screen contain canonical a GBS(s) within 1 kb of the peak (Figure 1C), and the average density of the GBS(s) present at the DamID peaks (0.693 GBS/kb) is significantly higher than the average GBS density over the whole genome (0.138 GBS/kb) (*P* = 1.496 × 10^−148^; Wilcoxon test = 0) (Figure 1D). Finally, because numerous GBSs are present throughout the genome but may not all be relevant to the Gcm cascade, we asked whether those that are under a DamID peak are more likely to be directly associated with Gcm. Indeed, the GBSs present under DamID peaks are significantly more conserved than the GBSs in the whole genome (12 *Drosophila* species, mosquito, honeybee, and red flour beetle were used for the comparative analysis), thus adding strength to the DamID data (*P* = 0.00273) (Figure 1E).

### The DamID screen identifies genes previously characterized as Gcm interactors

The 4863 DamID peaks are located in the vicinity of (<5 kb) or within 1031 genes (Figure 1F shows the overall peak locations). To assess the specificity of the DamID screen, known targets of Gcm were examined more closely. For instance, *gcm* itself contains several GBSs upstream of its transcription start site (TSS) and is known to autoregulate, and the strongest GBS was determined previously to be GBS 3/C, which is located 3 kb upstream of the *gcm* TSS (Miller *et al.* 1998; Ragone *et al.* 2003) (Figure 2A). A DamID peak was detected on top of this GBS (Figure 2A). Other examples include *sna*, *AGO1*, *brat*, and *lola*, which were all identified as *gcm* interactors in a genetic screen (Popkova *et al.* 2012) (Figure S1, A–D). All of them contain at least one significant DamID peak in their promoter regions. Moreover, the gene *loco* involved in late glial cell differentiation is controlled directly by Gcm (Granderath *et al.* 2000) and has five canonical GBSs, three of which are located within a DamID peak (Figure S1E). Importantly, the three GBSs located under the peak are critical for the expression pattern of *loco* in glial cell (Granderath *et al.* 2000). The gene *pnt*, involved in glial development (Chen *et al.* 1992; Klambt 1993) and the immune response (Zettervall *et al.* 2004), was described as downstream of *gcm* (Giesen *et al.* 1997) and contains one canonical GBS within one DamID peak (Figure S1F). Two other genes were extensively described as targets of Gcm during glial cell development: *ttk* and *repo*. Ttk is a transcriptional repressor inhibiting the neuronal fate in neural stem cells (Giesen *et al.* 1997). While containing two canonical GBSs within a DamID peak, *ttk* was not identified as a direct target of Gcm by our screen because the peak is located far (9.2 kb) from the TSS (Figure S1G). The Repo homeodomain transcription factor is required for the late differentiation of lateral glial cells (Campbell *et al.* 1994; Xiong *et al.* 1994; Halter *et al.* 1995) and is directly activated by Gcm through the 11 GBSs present in the 4.3-kb region upstream of the TSS (Lee and Jones 2005). However, *repo* was not selected in our screen because the observed DamID peak did not pass the enrichment threshold to be considered significant by the algorithm (Figure S1H). This indicates that the criteria for the identification of the Gcm direct targets are extremely stringent.

Three teams had previously performed genome-wide screens for the Gcm downstream targets (Egger *et al.* 2002; Freeman *et al.* 2003; Altenhein *et al.* 2006). Egger *et al.* (2002) compared WT stage 11 embryos to those expressing Gcm ectopically in the neuroectoderm (GOF embryos) and identified 356 genes significantly enriched in *gcm* GOF compared to WT animals (*P* < 0.001). Freeman *et al.* (2003) combined computational prediction, expression profiling analyses in WT and *gcm* GOF, and IHS in WT, *gcm* GOF, and *gcm* LOF animals to identify and validate 48 genes as downstream targets of Gcm. And finally, Altenhein *et al.* (2006) tracked Gcm downstream targets in stage 9–16 embryos in WT, *gcm* LOF, and *gcm* GOF animals and validated 119 genes by IHS. Together these studies identified 471 downstream targets of Gcm, but the overlap between the three data sets is quite weak, with only 42 genes identified by two of the studies and 5 genes identified by all three studies (Figure 2B). Cross-referencing the three data sets with the DamID peaks allowed us to considerably restrict the number of targets identified by the expression profiling analyses and revealed that 47 genes identified as downstream targets of Gcm in at least one of these studies are direct targets of Gcm according to DamID (Figure 2, C and D). Of note, in the first part of their study, Freeman *et al.* (2003) developed an algorithm to predict 384 direct targets of Gcm based on the presence of a cluster of eight GBSs in the surrounding regions. Among the predicted targets, only 8.3% (17 of 204 tested) were confirmed in Gcm GOF or Gcm LOF embryos (Freeman *et al.* 2003). Cross-referencing the DamID data set with these bioinformatics data returned 47 genes of 384 (12%) predicted to be direct targets by the Freeman *et al.* (2003) algorithm and confirmed as direct targets by our screen. Among these 47 genes, only 7 were previously validated *in vivo* (Table S1). Together with the observed evolutionary conservation of the GBSs under DamID peaks, this underlines the importance of scoring for occupied binding sites.

Finally, expression profiling and DamID analyses provide complementary information because the first approach tells the direct and indirect transcriptional consequences of a mutation, whereas the second tells where the transcription factor binds in the genome. We thus verified that the direct targets identified in our screen are differentially represented in the expression profiling data. Because expression of the direct targets is induced just after Gcm starts being expressed (stage 10), we expect them to be enriched in the expression profiling data relative to the early stages. We thus analyzed the data of Altenhein *et al.* (2006) and found that for most of the downregulated genes identified in the LOF expression profiling (119 in these data sets), expression starts being affected over a large time window, between stages 11 and 13 (Figure 2E). In contrast, when we performed the same analysis on the gene subset that also was detected in the DamID screen (13 genes), we found that most of these targets start to be downregulated at earlier stages (Figure 2F). Together these findings indicate that the DamID screen is an efficient method to identify the direct targets of Gcm.

### The DamID screen identifies new direct targets of Gcm

Among the 1031 genes identified by the DamID screen, more than 900 are new. The interaction between Gcm and four new target genes was validated by luciferase assays in *Drosophila* S2 cells. These genes were selected to be representative of the different locations of the DamID peaks. They include genes showing a DamID peak in the promoter and carrying canonical GBS-like *CG30002*; genes for which the closest GBSs are near the DamID peak but do not overlap with it, such as *CycA*; and genes for which the DamID peak and the GBSs are located within the transcribed region of the gene, such as *CycA*, *Enhancer of split m8* (*E(spl)m8*), and *ptc*. For each gene, the regions containing the GBS under the DamID peak or closest GBS to the DamID peak were cloned in a luciferase reporter plasmid. For DamID peaks covering two GBSs (*CG30002* and *ptc*), one reporter was built per GBS. The constructs then were transfected in S2 cells with or without the Gcm expression vector *ppacGcm*. In parallel, the same regions were mutated for their GBSs and analyzed similarly (Figure 3, A–D). The gene *CG30002* contains a significant DamID peak in its promoter region and two GBSs at the position of the peak (Figure 3A). The luciferase assays indicate that both GBSs induce transcription of the reporter on cotransfection with Gcm, and no induction is observed when the GBSs are mutated. Similar observations were made on *CycA*, *E(spl)m8*, and *ptc*, even though the DamID peaks are located within the coding sequences of these genes (Figure 3, B—D).

To confirm the data obtained with the reporter plasmids, we analyzed the effects of Gcm on the endogenous genes and measured the levels of their transcripts in S2 cells by qPCR. S2 cells were transfected with *ppacGcm* because Gcm is expressed at extremely low levels in those cells (Cherbas *et al.* 2011). We were able to show significant induction of *CycA* and *E(spl)m8* expression, but no induction was observed for *CG30002* or *ptc* (Figure 3E). Such a negative result might be due to the facts that S2 cells do not contain the appropriate cofactors, the genes are in a repressed chromatin state, and/or S2 cells have low transfection efficiency. Indeed, FACS analysis of S2 cells transfected with the Gal4 expression vector *ppacGal4* and UAS-*GFP* vectors revealed that only 4.92% of the cells express GFP. This means that only a minority of the cell population contains the two plasmids (Figure 3, G and H). To improve the readout of the assay, S2 cells were transfected with *ppacGcm* and the reporter plasmid *repoGFP*, which was used previously to trace Gcm activity (Lee and Jones 2005; Laneve *et al.* 2013; Flici *et al.* 2014). This allowed us to sort the cells that express the GFP produced under the *repo* promoter. Based on the FACS analysis, our transfection protocol allows the detection of GFP in 2.11% of S2 cells (Figure 3I). These GFP^+^ cells were enriched to reach at least 80% purity, and the preceding target genes were analyzed by qPCR. First, we monitored the levels of *repo* endogenous transcripts to assess the efficiency of our protocol. Without FACS sorting, no change in *repo* levels was observable on Gcm expression, whereas we could see a 30-fold increase on adding the FACS sorting step (Figure 3F). This step also allowed us to detect the induction of *CG30002* and *ptc* expression (Figure 3F) and greatly improved the detection of *E(spl)m8* and *CycA* transcript induction.

**Figure 4:**
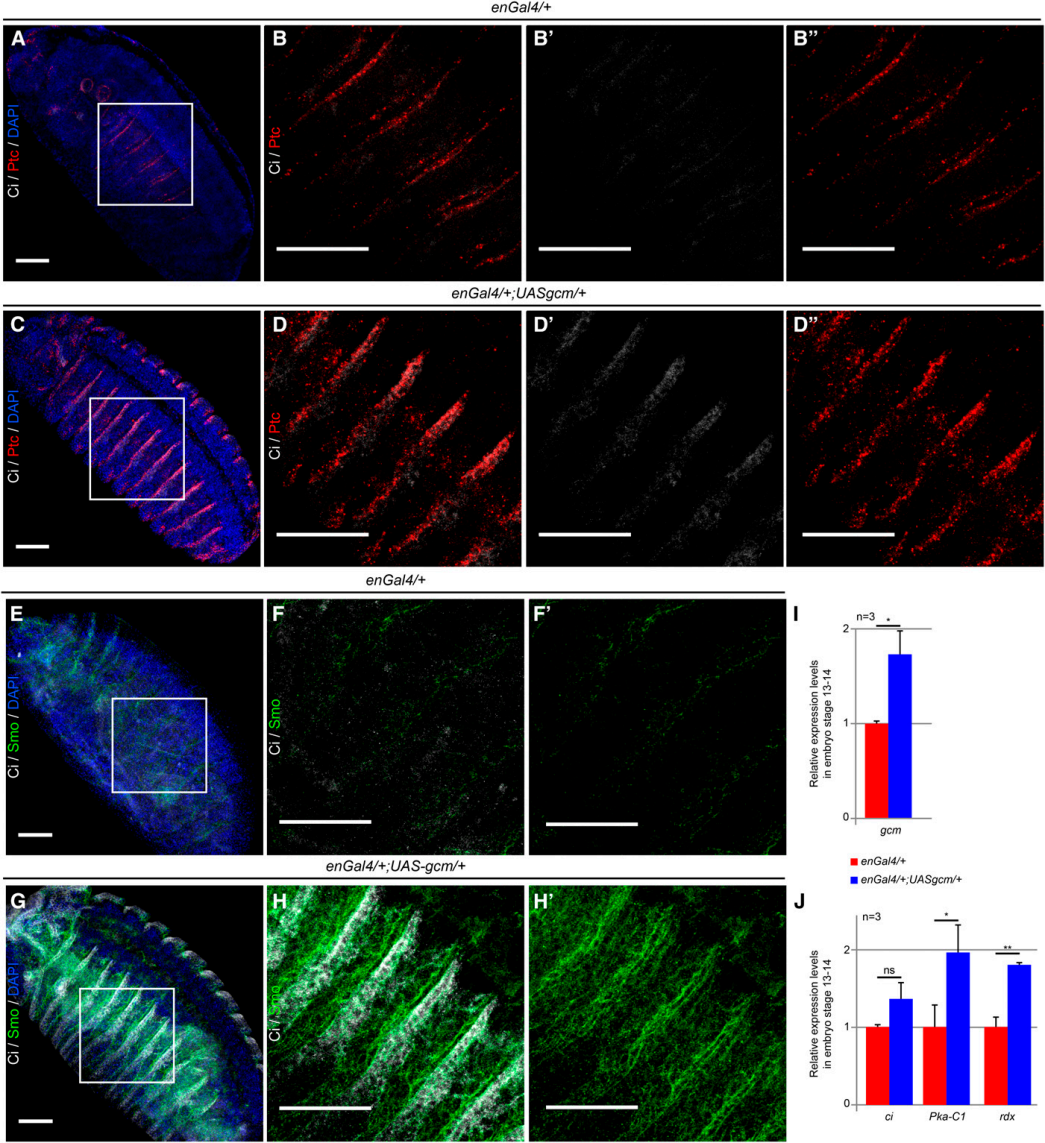
Gcm regulates the Hedgehog signaling pathway in the embryonic epidermis. (A–H) Immunolabeling of Ci (in gray), Ptc (in red), and Smo (in green) in stage 15 embryos of the following genotypes: *enGal4*/*+* (control) and *enGal4*/*+*; *UASgcm*/*+* (*gcm* GOF). The areas delimited in white in A, C, E, and G are magnified in B–B″, D–D″, F and F′, and H and H′, respectively. Full projections of the embryos are shown in A, C, E, and G, and projections of four optical sections taken at 2-µm interval are shown in the magnified regions of B, D, F, and H. DAPI-labeled nuclei are in blue. Note that the three proteins involved in the Hedgehog signaling pathway are expressed at higher levels in the *gcm* GOF than in the control embryo. Bar, 50 µm. (I and J) Histograms showing the expression of *gcm* (I), *ci*, *Pka-C1*, and *rdx* (J) in *enGal4*/*+* (control, red) and *enGal4*/*+*; *UASgcm*/*+* (blue) embryos at stage 13–14. The *y*-axis represents the relative expression levels compared to that observed in the control embryos (red columns). Error bars and the *P*-values are calculated as in Figure 3D.

Finally, we tested DamID target genes *in vivo*. Because *ptc* is strongly required in the epidermis at the level of muscle attachment sites, where Gcm is also expressed and required, we drove epithelial Gcm expression using the *engrailedGal4* (*enGal4*) driver (Figure 4I), which induces expression of tendon cell markers (Soustelle *et al.* 2004). Because several other members of the Hh pathway also were identified in the DamID screen, including *rdx*, *smo*, *ci*, and *Pka-C1*, we analyzed these genes as well. First, we performed qPCR assays and found increased expression for some of them (*Pka-C1* and *rdx*) (Figure 4J). To complement this approach, we performed qualitative analyses by immunolabeling on Gcm-overexpressing embryos and found significantly increased expression for Ptc, Ci, and Smo (Figure 4, A–H′). In agreement with previously obtained data (Soustelle *et al.* 2004), the expression of Repo did not increase, likely owing to the lack of cell-specific factors, which are known to affect Gcm activity strongly (De Iaco *et al.* 2006). Conversely, the expression of Repo increases on overexpression of Gcm in the neurogenic region, whereas that of Ci, Ptc, and Smo does not (data not shown). In sum, the *in vivo* findings validate those in S2 cell transfection assays, which thus provide a simple and sensitive approach.

**Figure 5:**
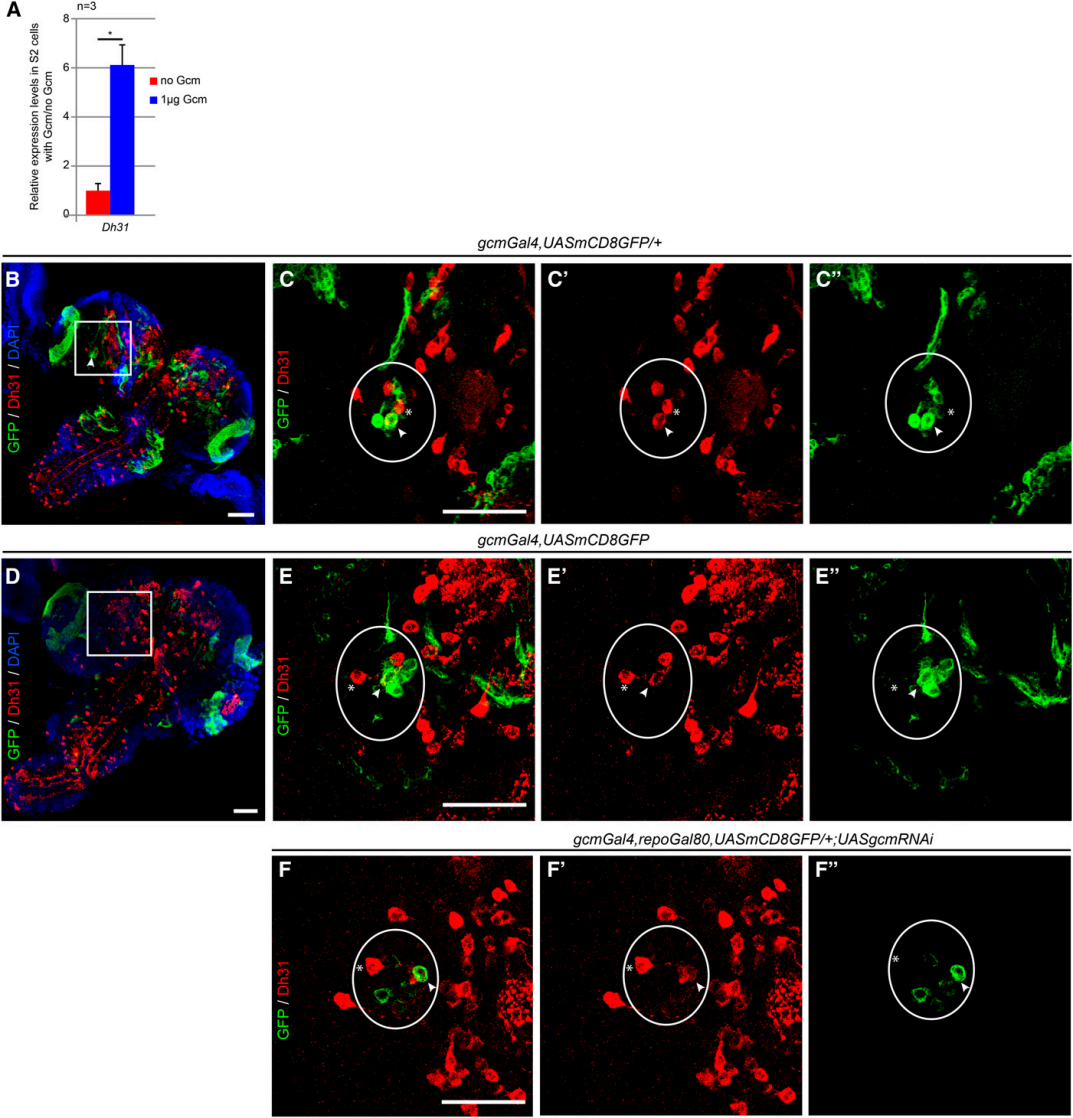
Gcm overlaps with and regulates Dh31 expression in the larval CNS. (A) Histogram representing the endogenous expression levels of Dh31 in S2 cells with and without transfected Gcm. Error bars and *P*-values are calculated as in Figure 3, A–D. (B–F″) Immunolabeling of Dh31 and GFP in larval CNS (Dh31 in red; GFP in green). White arrowheads indicate cells coexpressing Dh31 and Gcm (GFP^+^ cells); asterisks indicate cells expressing only Dh31. The *gcm*-expressing cells correspond to the dorsolateral neuronal cluster. Areas delimited in white in B and D are magnified in C–C″ and E–E″, respectively. (B) Full projection of a larval CNS of a heterozygous *gcmGal4*, *UASmCD8GFP* at the third instar. (C–C″) Projection of three optical sections taken at 2-µm interval: (C) overlay of Dh31 and GFP, (C′) Dh31 alone, and (C″) GFP alone. (D–E″) Same as B–C″ in a *gcmGal4*, *UASmCD8GFP* homozygous larva. (F–F″) Same as C–C″ in a *gcm* knockdown (KD*)* larva of the following genotype: *gcmGal4*, *repoGal80*, *UASmCD8GFP*/*+*; *UASgcmRNAi*/*+*. In all genotypes, the three sections contain the whole dorsolateral cluster (white oval). Note that in the *gcmGal4* homozygous and in the *gcm* KD (arrowheads) larvae, the intensity of Dh31 labeling is reduced in the GFP^+^ cell compared to that observed in the control *gcmGal4* heterozygous animals. Also take for comparison the surrounding Dh31^+^ cells (asterisk). (B and D) DAPI staining shows the nuclear labeling is in blue. Bar, 50 µm.

Moreover, the targets identified by the DamID screen are not necessarily expressed in embryos at stage 11, the stage at which the screen was performed. Indeed, comparison of DamID and modENCODE transcriptome data on stage 10–11 embryos reveals that 8.7% of the genes identified in our screen are not expressed at this stage (Figure 3J). For example, the *Diuretic hormone 31* (*Dh31*) gene is not detected in embryos on ISH (Tomancak *et al.* 2002, 2007; Hammonds *et al.* 2013) and starts to be expressed at stage 17 according to modENCODE data (Graveley *et al.* 2011). Nevertheless, the *Dh31* locus contains two DamID peaks, and Gcm induces Dh31 expression in S2 cells (Figure 5A). We therefore analyzed later developmental stages and found colocalization of Dh31 and Gcm in a single cell of the larval brain hemisphere (Figure 5, B–C″). At that stage, Gcm is expressed in the *de novo*–produced glial cells of the optic lobe, in the lamina, and in medulla neurons (Chotard *et al.* 2005), as well as in two groups of neurons of the central brain, the so-called dorsolateral and medial clusters (Soustelle *et al.* 2007). The double Gcm/Dh31^+^ cell belongs to the dorsolateral cluster, and colocalization is affected in *gcm* mutant animals. The *gcmGal4*, *UASGFP* line allowed us to trace *gcm* expression in WT and hypomorphic conditions obtained by using *gcmGal4* homozygous or heterozygous animals carrying a *UASgcmRNAi* construct (Figure 5, D–F). These data strongly suggest that the Dam construct is present and can bind sites in cells in which Gcm and/or its targets are not yet expressed. In sum, we have shown that S2 cells can be used to validate direct targets of Gcm on cell sorting, that the identified targets are actually induced by Gcm *in vivo*, and that the Dam-Gcm fusion likely identifies most Gcm direct targets in the fly genome.

### Protein domain enrichment analysis

**Figure 6:**
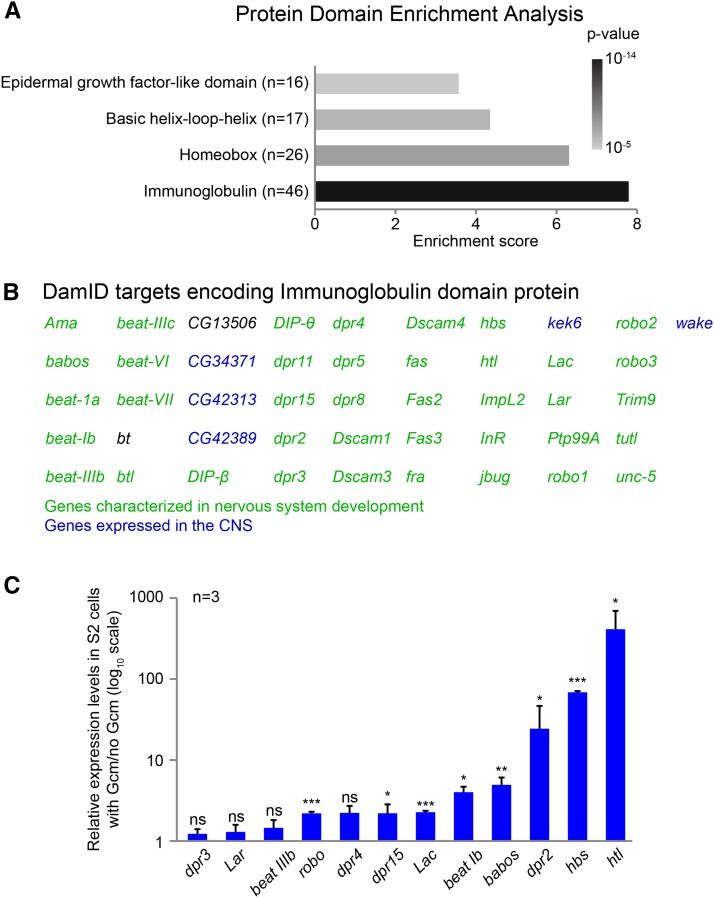
Gcm targets encode Ig domain proteins. (A) Histogram summarizing the protein domain enrichment analysis. The *x*-axis indicates the enrichment score; the grade of gray is representative of the *P*-value: lightest gray, *P* = 10^−5^; black, *P* = 10^−14^. The *y*-axis indicates the name of the protein domain; *n* indicates the number of genes in the DamID screen containing that domain. (B) List of genes from the DamID screen containing immunoglobulin (Ig) domains. The genes indicated in green are known to be involved in nervous system development; the genes in blue are expressed in the nervous system. (C) Histogram showing the endogenous expression of Ig domain–containing genes on S2 cell transfection with a Gcm expression vector and FACS sorting. The *y*-axis represents the relative expression levels in cells transfected with Gcm compared to cells without Gcm. The *y*-axis is in log_10_ scale; error bars and *p*-values are calculated as in Figure 3D.

To annotate the genes identified in the DamID screen, we first performed a protein domain enrichment analysis using DAVID bioinformatics resources (Huang *et al.* 2009a,b). The analysis showed enrichment for genes coding for proteins containing basic Helix-Loop-Helix (bHLH) domains (17 genes) and Homeobox domains (26 genes), which are characteristic of transcription factors, but the most enriched family is the Ig domain–containing protein (DAVID protein domain enrichment: 7.79-fold; *P =* 1.3 × 10^−14^; FDR = 2.0 × 10^−11^) (Figure 6A). Most of the Ig genes are involved in or at least expressed during nervous system development (Figure 6B) and code for guidance molecules [reviewed in Patel and Van Vactor (2002)]. This includes two fibroblast growth factor receptors (Htl and Btl), two netrin receptors (Unc-5 and Fra), six members of the Beaten path family (Beat), three members of the Down syndrome cell adhesion molecule family (Dscam), two fasciclins (Fas), three roundabout proteins (Robo), and several others, including seven members of the defective proboscis extension response family (Dpr) and two Dpr interactors (Ozkan *et al.* 2013) (Figure 6B and Table 1). The chemoreceptor family of genes *dpr* has been poorly characterized so far, but these genes are also expressed in glial cells (DeSalvo *et al.* 2014), suggesting that Gcm may regulate these genes during gliogenesis.

To confirm induction of this class of genes by Gcm, the levels of expression of 12 of them were assayed in S2 cells in basal conditions or on transfection with a Gcm expression vector (Figure 6C). The expression of 8 genes of 12 is significantly induced by Gcm in S2 cells, the strongest induction being observed for *htl* (>100-fold increase) (Figure 6C).

### GO term enrichment analysis

#### Gcm direct targets are involved in nervous system development:

**Figure 7:**
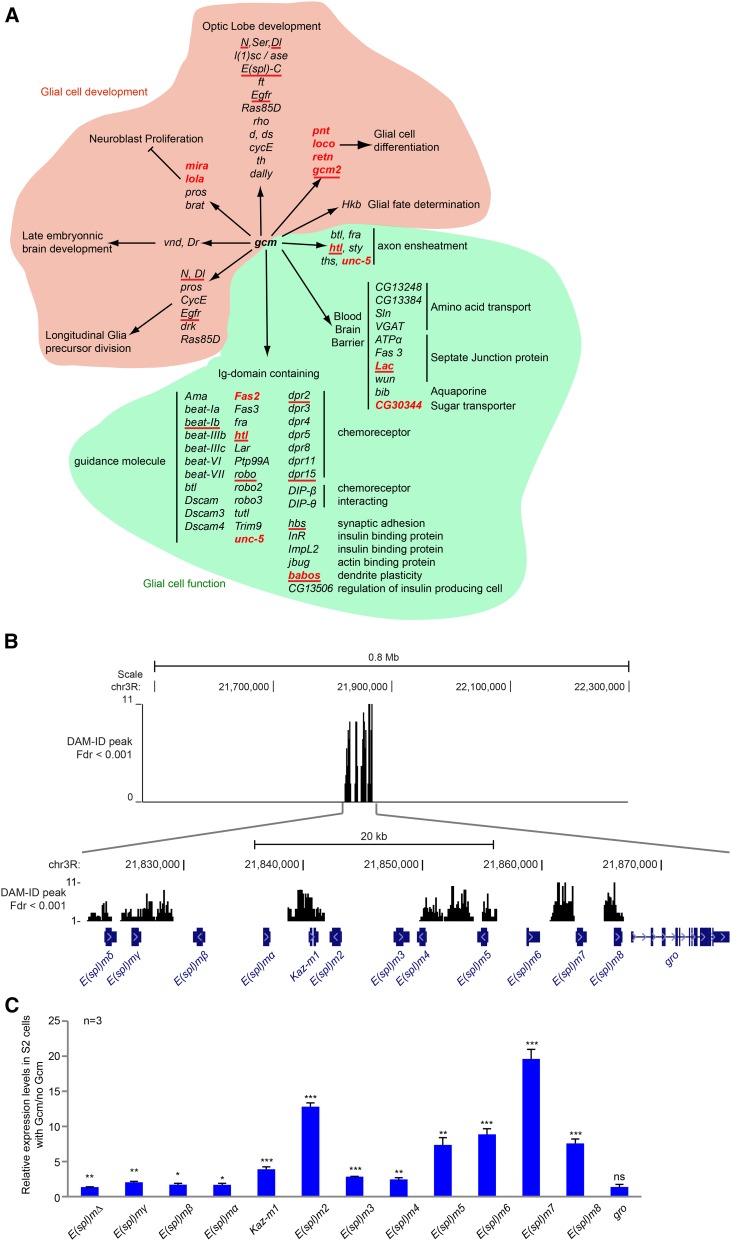
Gcm targets involved in nervous system development and function. (A) List of DamID genes involved in neural development and function. The genes in red were previously characterized as downstream targets of Gcm, and those underlined were confirmed by qPCR in FACS-sorted S2 cells in this study. (B) Schematic representation of *E(spl)-C*. Top panel represents the DamID peak histogram on the 0.8 Mb around *E(spl)-C*. Note that the peaks are all localized within *E(spl)-C*. Bottom panel shows the 50-kb window of *E(spl)-C*. (C) Histogram represents the S2 cell endogenous levels of *E(spl)-C* transcripts on transfection of a Gcm expression vector, as indicated in Figure 3, A–D.

Following protein domain enrichment analysis, we carried out a GO term enrichment analysis for biological function using DAVID. The analysis retrieved 230 genes involved in nervous system development (8.8-fold enrichment; *P =* 2.4 ×10^−12^; FDR = 3.9 × 10^−9^). This subset of genes was then further analyzed using an enrichment analysis for molecular function. As expected from a cell fate determinant, the major class of genes regulated by Gcm and involved in nervous system development is transcription factors (67 genes; 6.7-fold enrichment; *P =* 1.0 × 10^−32^; FDR = 1.3 × 10^−29^) (list in Table S1, column J). More specifically, we found genes involved in (1) neural stem cell (also called *neuroblast*) regulation, (2) embryonic glial cell development, and (3) larval optic lobe development (Figure 7A and Table 1).

1. Up to 34 genes regulate neural stem cells, including genes that likely allow the transition from stem cell to glial identity. Interestingly, Pros also was identified as a positive regulator of Gcm (Freeman and Doe 2001; Ragone *et al.* 2001; Choksi *et al.* 2006), and both Brat and Lola interact with Gcm genetically (Popkova *et al.*, 2012). These data suggest the presence of feedback loops in the establishment of glial fate.
2. Numerous targets are directly linked to glial cell development, as expected given the gliogenic role of Gcm. For example, *Hkb* controls glial subtype specification by reinforcing Gcm autoregulation in a specific glial lineage and interacts with Gcm genetically as well as biochemically (De Iaco *et al.* 2006) (Popkova *et al.* 2012). In addition, several genes are required in longitudinal glia precursor division (Figure 7A and Table 1).
3. As to genes involved in optic lobe development, this is in line with the finding that Gcm is necessary for both neuronal and glial cell development within the larval optic lobe (Chotard *et al.* 2005; Yoshida *et al.* 2005; Soustelle *et al.* 2007). Of note, one of the targets, Tll, has been shown recently to be necessary for specification of lamina neuronal precursors, showing a mutant phenotype similar to that induced by the lack of Gcm (Guillermin *et al.* 2015).

**Figure 8:**
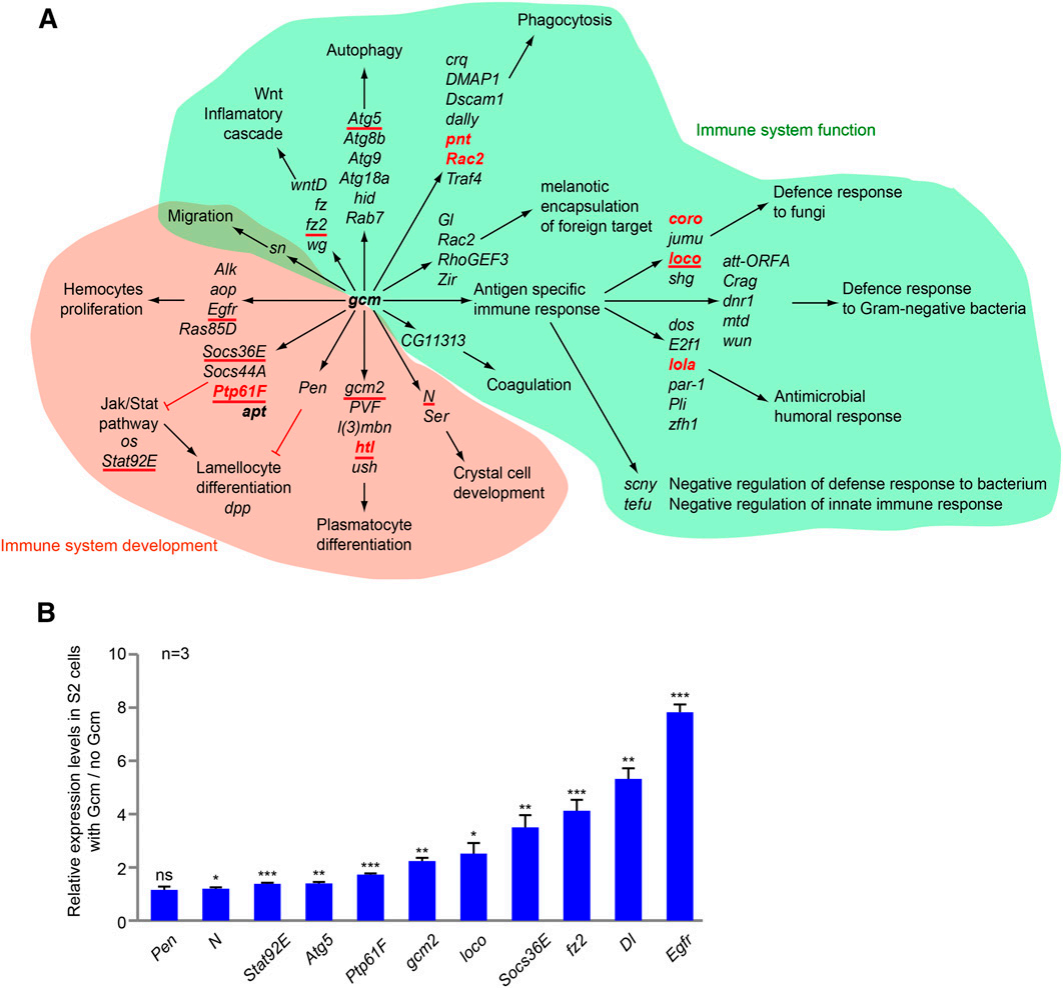
Gcm targets involved in immune system development and function. (A) List of the DamID genes. Green area covers those involved in immune system function; red area covers those involved in immune system development. The genes in red were previously characterized as downstream targets of Gcm, and those underlined were confirmed by qPCR in FACS-sorted S2 cells in this study. (B) Histogram representing the S2 cell endogenous levels of genes involved in immune system development on transfection with a Gcm expression vector, as indicated in Figure 3, A–D.

Among the targets, we identified several members of signaling pathways that control neural development at different steps: Hh, Egfr/Ras, and Fat/Hippo pathways (*ft*, *Egfr*, *Ras85D*, *rho*, *ds*, *d*, *CycE*, *th*, and *dally*) and, finally, the N pathway (*N*, *Ser*, *Dl*, *l(1)sc*, *ase*, and eight genes of the *E(spl)* complex, or *E(spl)-C*). Because of their peculiar organization, we further validated the genes of *E(spl)-C*, which spans over 50 kb and is located on the right arm of chromosome *3* (Figure 7, B and C). Its members are all induced in FACS-sorted S2 cells transfected with Gcm, whereas the gene directly adjacent to the complex, *gro*, is devoid of a DamID peak and is not induced, indicating that Gcm activity is specific to the complex (Figure 7C). *N* and *Dl* also were validated (Figure 8B).

Interestingly, several genes targeted by Gcm are involved in the function of fully differentiated glia. Our screen identified several genes acting in the blood-brain barrier (BBB) (Figure 7A and Table 1). Overlap between our screen and transcriptome data of the BBB reveals that Gcm targets 61 genes enriched in the BBB compared to all glial cells (Table S1, column L) (DeSalvo *et al.* 2014; Limmer *et al.* 2014). Gcm also targets several genes controlling axon ensheathment and glial cell migration (Figure 7A and Table 1). In sum, the screen reveals the molecular role and mode of action of Gcm in neural development and function.

#### Gcm direct targets are involved in immune system development:

In addition to its role in the nervous system, Gcm is also required for differentiation and proliferation of embryonic plasmatocytes. Embryos mutant for *gcm* and its paralog *gcm2* present a decreased number of plasmatocytes, and the plasmatocytes do not complete the differentiation process (Bernardoni *et al.* 1997; Alfonso and Jones 2002) [reviewed in Kammerer and Giangrande (2001), Evans and Banerjee (2003), and Waltzer *et al.* (2010)]. Plasmatocytes are macrophages that can differentiate into another type of hemocyte called a *lamellocyte* on immune challenge (Rizki 1957; Stofanko *et al.* 2010), a process that Gcm helps to repress (Jacques *et al.* 2009). The DamID screen identified 68 genes known to regulate the immune system (Figure 8A, Table 2, and Table S1, column M), and similar to the nervous system, transcription factors are quite prominent (14 transcription factors) (Table S1, column J).

In addition to *gcm* itself and *gcm2* (Bernardoni *et al.* 1997; Kammerer and Giangrande 2001; Alfonso and Jones 2002), Gcm targets genes that promote the differentiation of prohemocytes into plasmatocytes (Figure 8A and Table 2). Several targets inhibit the JAK/STAT pathway, whose activation leads to lamellocyte differentiation (Figure 8A and Table 2). *Pen* and *ush* inhibit the formation of lamellocytes and so-called melanotic tumors, which are masses of aggregated hemocytes enriched in lamellocytes (Kussel and Frasch 1995; Sorrentino *et al.* 2007). In addition, Gcm targets genes characterized by their role in hemocyte proliferation or migration (Figure 8A and Table 2).

Similar to the nervous system, Gcm also targets genes that are involved in function of the mature immune system. These include genes involved in (1) coagulation, (2) phagocytosis, (3) autophagy, (4) the inflammatory cascade mediated by Wnt, and (5) melanotic encapsulation of foreign targets (Figure 8A and Table 2). Finally, DamID targets also include genes that tune the immune response based on the nature of the pathogen. Thus, based on the screen, Gcm induces the expression of genes involved in the response to fungi, in the response to gram-negative bacteria, and more broadly, in the antimicrobial humoral response (Figure 8A and Table 2). In addition, we assayed 11 genes involved in hemocyte biology using S2 cells transfected by a Gcm expression vector and confirmed the induction of 10 of them (Figure 8B).

#### Gcm direct targets are involved in tendon cell and peritracheal cell development:

Tendons cells link muscles to the backbone of the organism, and Gcm expression in these cells is required for proper muscle attachment (Soustelle *et al.* 2004). Several Gcm targets are involved in the maturation of tendon cells, such as Hh signaling pathway proteins Wg and Egfr. The Hh signaling pathway and Wg are necessary in the early development of tendon cells (Hatini and DiNardo 2001), whereas the Egfr pathway promotes terminal differentiation after the junction between the migrating muscle and the tendon has been established (Yarnitzky *et al.* 1997).

Other targets expressed in tendon cells are directly involved in muscle migration toward the tendon. Sli and Sdc are guidance cues that attract the muscle (Kramer *et al.* 2001; Steigemann *et al.* 2004). Leucine-rich tendon-specific protein (Lrt) interacts with Robo expressed in the muscle to arrest muscle migratory behavior (Wayburn and Volk 2009). *Dnt* and *Hbs* control muscle attachment site selection (Dworak *et al.* 2001; Lahaye *et al.* 2012). A third class of targets controls the later step of building the junction between muscles and tendons. Mew, If, Dys, and Wech are core components of the junction (Prokop *et al.* 1998; Loer *et al.* 2008) [reviewed in Charvet *et al.* (2012)]; Short stop (Shot) is an actin-tubulin cross-linker involved in junction stabilization (Bottenberg *et al.* 2009), and Sema-5C is a transmembrane protein so far poorly characterized in tendon cells (Bahri *et al.* 2001).

Finally, Gcm is expressed in peritracheal cells (Laneve *et al.* 2013), endocrine cells located along the trachea that secrete ecdysis-triggering hormone (O’Brien and Taghert 1998). Only two markers of peritracheal cells have been characterized so far: the bHLH transcription factor Dimm (Hewes *et al.* 2003) and the neuropeptide biosynthetic enzyme peptidylglycine-α-hydroxylating monooxygenase (Phm) (O’Brien and Taghert 1998). Gcm acts upstream of *dimm* (Laneve *et al.* 2013), and *dimm* is known to control the expression of *phm* (Park *et al.* 2008), but neither *dimm* nor *phm* is present in the DamID screen, suggesting that Gcm does not directly induce these genes in peritracheal cells. While little is known about peritracheal cells, *dimm* and *phm* were further characterized in neuroendocrine cells: 212 downstream targets of Dimm were recently identified by combining CHiP and transcriptome analyses (Hadzic *et al.* 2015). Comparison of this data set with the DamID screen revealed that 27 targets are common to Dimm and Gcm (Table S1), suggesting a potential contribution of Gcm to the Dimm regulatory pathway. In addition, based on our screen, Gcm targets a gene involved in the endocrine function of Dimm^+^ cells: *syt-β* is controlled by Dimm and is involved in calcium-dependent exocytosis (Adolfsen *et al.* 2004; Park *et al.* 2014). To conclude, Gcm is necessary for the development of peritracheal cells expressing Dimm, and our screen identified genes involved in the Dimm pathway. This sets the stage for future analysis of these genes in peritracheal cells. Overall, our screen reveals the full extent of Gcm function in the diverse cell types in which this transcription factor is expressed.

### Conservation of the Gcm molecular cascade in mammals

The mammalian genome contains two orthologs of *Drosophila gcm* genes, which are named in humans *GCM1* and *GCM2*. *GCM1* is required for differentiation of trophoblasts in the developing placenta (Basyuk *et al.* 1999, 2009); *GCM2* is expressed and required mainly in the parathyroid glands (Kim *et al.* 1998; Gordon *et al.* 2001). Because few targets have been identified for GCM1 and GCM2, we sought for a conserved Gcm regulatory cascade on retrieving the mammalian orthologs of the *Drosophila* targets identified in the DamID screen (Table S1). To start a comparative analysis, we chose genes that have a functional significance in mammals. A GO term enrichment analysis identified orthologs that are associated with parathyroid gland or placenta development, which allowed us to restrict the list to 29 genes potentially targeted by *GCM* genes in mammals (Table S2). We further analyzed the impact of murine Gcm genes on *GCM1* and *GCM2*; *T-box transcription factor* (*TBX1*); *GATA3*, *GATA4*, and *GATA6*; *FGFR1* and *FGFR2*; and *Delta-like 1* (*DLL1*) expression.

### GCM genes regulate their own expression

**Figure 9:**
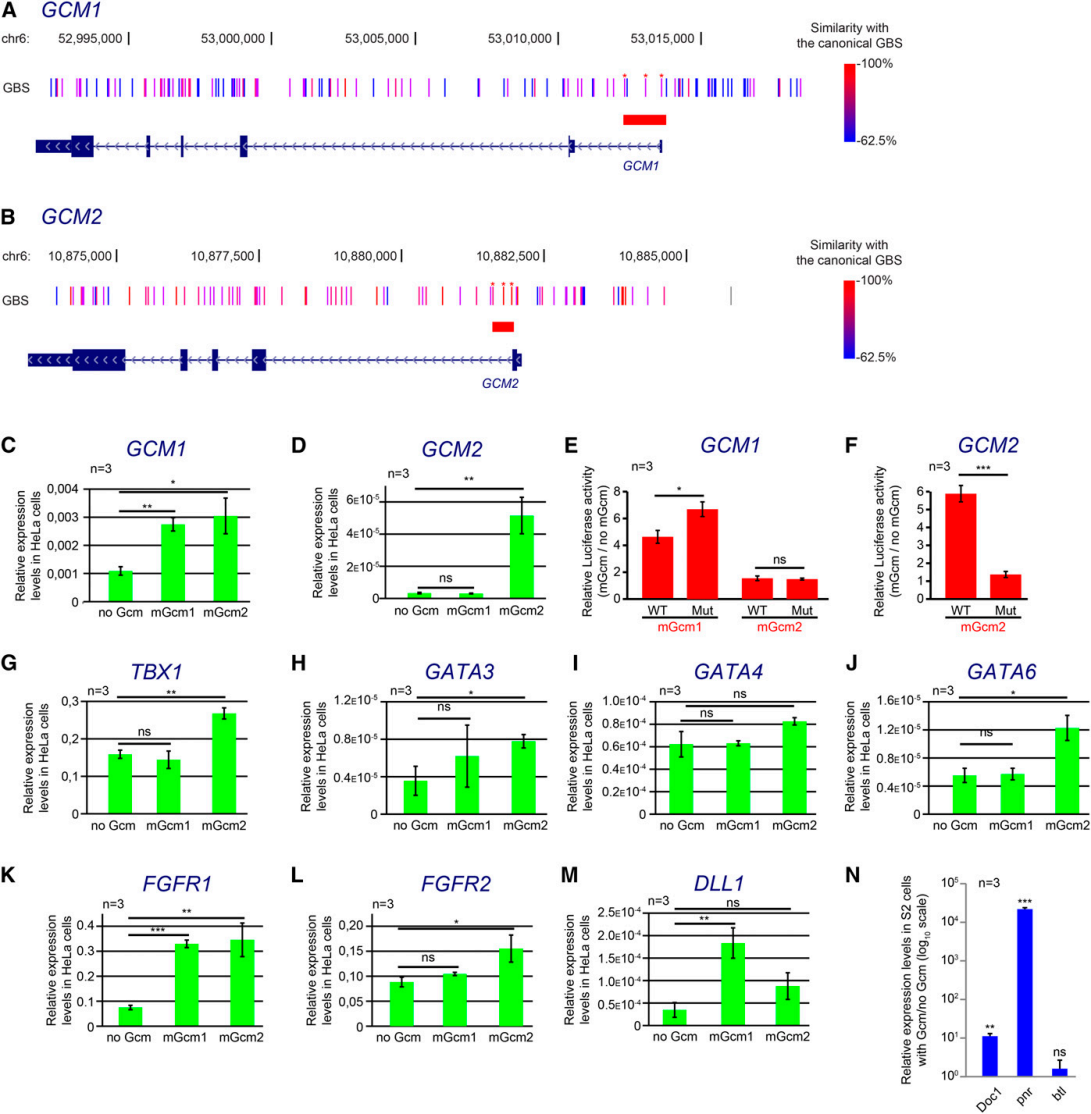
Conservation of Gcm targets in mammals. (A and B) Schematic representation of *GCM1* (A) and *GCM2* (B) loci in humans. The genes are represented as in Figure 2A. GBSs are indicated as bars. The color coding refers to their similarity with the canonical GBSs used in mammals in Yu *et al.* (2002) from 62.5% (blue) to 100% (red). The genomic coordinates of the loci (genome version GRCh37/hg19) are indicated above the GBSs. Red rectangles indicate the regions used for the luciferase reporter assays, and the mutated sites are indicated by red asterisks. (C and D) Characterization of mGcm1 and mGcm2 effects on *GCM1* (C) and *GCM2* expression (D). Histograms represent the endogenous expression levels of transcripts of each gene in HeLa cells on transfection with mGcm1 or mGcm2; values are relative to those of housekeeping genes *GAPDH* and *ACTB*. (E) Histogram representing the luciferase activity in HeLa cells transfected with mGcm1 or mGcm2 and luciferase reporters containing the region of GCM1 with WT or mutant GBSs. Levels of luciferase activity are relative to those observed in the absence of mGcm transfection. (F) Histogram representing the luciferase activity in HeLa cells transfected with mGcm2 and luciferase reporters containing the region of GCM2 with WT or mutant GBSs as in E. (G–M) Characterization of mGcm1 and mGcm2 effects on *TBX1* (G), *GATA3* (H), *GATA4* (I), *GATA6* (J), *FGFR1* (K), *FGFR2* (L), and *DLL1* expression (M), as in C and D. (N) Histogram representing the S2 cell endogenous levels of *Doc1*, *pnr*, and *btl* on transfection with a Gcm expression vector, as indicated in Figure 3D.

*GCM1* and *GCM2* contain multiple GBSs in their promoters (Figure 9, A and B). This suggests the existence of a positive-feedback loop that has not been documented in mammals but that is very well characterized for *Drosophila gcm* genes (Miller *et al.* 1998; Ragone *et al.* 2003). To validate the autoregulation of GCM1 and GCM2 in mammals, we monitored their levels of expression in HeLa cells transfected with expression vectors for mGcm1 and mGcm2. The use of mouse orthologs allowed us to design qPCR primers specific for the human transcripts and to specifically quantify their levels of expression. This set of experiments shows that in HeLa cells, *GCM1* expression is induced by both mGcm1 and mGcm2 (Figure 9C), and *GCM2* is induced only by mGcm2 (Figure 9D).

To demonstrate that the induction of *GCM1* and *GCM2* expression was carried out via the GBSs, we arbitrarily selected promoter fragments containing three GBSs with at least 75% similarity with the canonical GBS ATG(A/C)GGG(T/C) (Yu *et al.* 2002). These regions were cloned in luciferase reporters (Figure 9, A and B, red). For each region, we also built the reporter with the mutated GBSs. The reporters were then transfected in HeLa cells with or without mGcm1 or mGcm2. For *GCM1*, we could observe an induction by *mGcm1* and not by *mGcm2*, suggesting that the region inserted in the reporter allows for Gcm1-mediated induction. However, the three GBSs are not sufficient for induction mediated by mGcm1 because their mutagenesis leaves it unaffected (Figure 9E). Further scrutiny of the region revealed the presence of two other GBSs with <75% similarity with the canonical GBS but still containing nucleotides 2, 3, 6, and 7, which were determined to be indispensable for Gcm binding (Schreiber *et al.* 1998). For *GCM2*, we showed that this gene is able to induce expression of the luciferase reporter carrying the *GCM2* promoter and that this promoter is inactive when the canonical GBSs are mutated (Figure 9F). This demonstrates that GCM2 is able to regulate its own expression via the GBSs inserted in the luciferase reporter. To conclude, mGcm1 and mGcm2 induce *GCM1* expression via a region that has to be defined, and mGcm2 activates *GCM2* transcription via a region covering the first exon-intron junction. These experiments demonstrate that positive autoregulation is conserved between *Drosophila* and mammalian *gcm* genes.

### Other DamID targets are conserved in mammals

*TBX1* was shown previously to be coexpressed with *GCM2* during formation of the parathyroid glands (Manley *et al.* 2004; Reeh *et al.* 2014) and contains 46 GBSs in its promoter. To assess whether the GCM transcription factors are able to induce *TBX1* expression in mammals, we analyzed the levels of *TBX1* transcripts in HeLa cells transfected with expression vectors coding for mouse orthologs mGcm1 or mGcm2. This assay indicated that expression of *TBX1* is specifically induced by mGcm2 (Figure 9G).

The three GATA transcription factors GATA-3, GATA-4, and GATA-6 control numerous developmental processes in mammals [reviewed in Molkentin (2000), Cantor and Orkin (2005), Zaytouni *et al.* (2011), and Chlon and Crispino (2012)]. The three genes contain several GBSs in their promoters, and expression of both *GATA3* and *GATA6* is induced by mGcm2 in HeLa cells (Figure 9, H–J).

*FGFR1* and *FGFR2* are the mammalian orthologs of the DamID targets *btl* and *htl*. FGFRs are widely described for their roles in angiogenesis, cancer development, and organogenesis [reviewed in Bates (2011), Kelleher *et al.* (2013), and Katoh and Nakagama (2014)]. They are also required for building the placental vascular system and are expressed in trophoblasts (Anteby *et al.* 2005; Pfarrer *et al.* 2006). Both genes contain GBSs in their promoters. In HeLa cells, mGcm1 induces the expression of *FGFR1*, and mGcm2 induces the expression of both *FGFR1* and *FGFR2* (Figure 9, K and L).

Finally, *DLL1* is one of the ligands of the N receptor (Shimizu *et al.* 2000). The N signaling pathway plays a critical role in cell fate determination [reviewed in Artavanis-Tsakonas *et al.* (1999) and Schwanbeck *et al.* (2011)], including trophoblast development (Zhao and Lin 2012; Rayon *et al.* 2014; Massimiani *et al.* 2015), and *DLL1* is expressed in trophoblasts (Herr *et al.* 2011; Gasperowicz *et al.* 2013). In humans, the *DLL1* promoter contains GBSs, and *DLL1* expression is induced specifically by mGcm1 in HeLa cells (Figure 9M).

### The *Drosophila* orthologs of mammalian target genes work in pathways known to depend on Gcm

The preceding data indicate that TBX1, GATA factors, the FGFRs, and the N ligand DLL1 are regulated by Gcm in mammals. As mentioned earlier, no tissue has been found so far for which Gcm function is required in both mammals and *Drosophila*. This suggests that instead of a conservation of Gcm in similar tissues, we should look for conserved Gcm cascades. To further test this hypothesis, we validated the impact of *Drosophila* Gcm transcription factor on the *Drosophila* orthologs of the four families of genes identified earlier.

The *Drosophila* orthologs of TBX1 are the T-box transcriptions factors Bi, H15 (Porsch *et al.* 1998), and Doc1, which are required for ganglion mother cell (GMC) differentiation during development of the embryonic nervous system (Choksi *et al.* 2006; Leal *et al.* 2009). We validated the role of Gcm on *Doc1* in S2 cells (Figure 9N).

The *Drosophila* ortholog of the GATA factor Pnr regulates postembryonic tendon cell differentiation (Ghazi *et al.* 2003). There is a significant induction of *pnr* expression by Gcm in S2 cells (Figure 9N), but further experiments are required to demonstrate the impact of Pnr on embryonic tendon cells or the impact of Gcm on postembryonic tendon cells.

The ortholog of the FGFRs, *htl*, is involved in ensheathing glia morphogenesis (Stork *et al.* 2014), and Gcm is required for differentiation of these cells (Awasaki *et al.* 2008). Gcm strongly induces expression of *htl* in S2 cells (Figure 6C). The second ortholog, *grn*, presents the same expression pattern as *gcm* in the developing embryonic CNS at stage 11 (Lin *et al.* 1995), indicating that Gcm may regulate *grn* expression in this tissue.

Finally, the DLL1 *Drosophila* ortholog Dl is required as part of the N pathway for the development of embryonic glia (Udolph *et al.* 2001; Van de Bor and Giangrande 2001; Umesono *et al.* 2002; Edenfeld *et al.* 2007), the larval optic lobe (Egger *et al.* 2010; Yasugi *et al.* 2010; Wang *et al.* 2011), and tendon cells (Nabel-Rosen *et al.* 1999; Ghazi *et al.* 2003). *Dl* expression is induced by Gcm in S2 cells (Figure 8B).

## Discussion

The DamID approach allowed us to identify the direct targets of Gcm in *Drosophila* in a genome-wide fashion and to extend these findings to mammals. It also allowed us to recognize key molecular pathways and developmental processes that depend on Gcm. The improvement of the transfection assays on FACS sorting provides a rapid and sensitive tool to characterize molecular pathways and genetic interactions. This versatile approach is particularly useful to study genes that are expressed at weak levels, in very few cells, or for which the target tissues are still unknown.

### Feedback loops between Gcm and signaling pathways

Gcm is widely described for its role in nervous system development and hemocyte differentiation, and indeed, many of the genes identified in the screen are involved in these two processes (Table 1 and Table 2). The screen allowed us to establish a direct link between Gcm and three major signaling pathways, the vast majority of whose members are targeted by Gcm.

**Figure 10:**
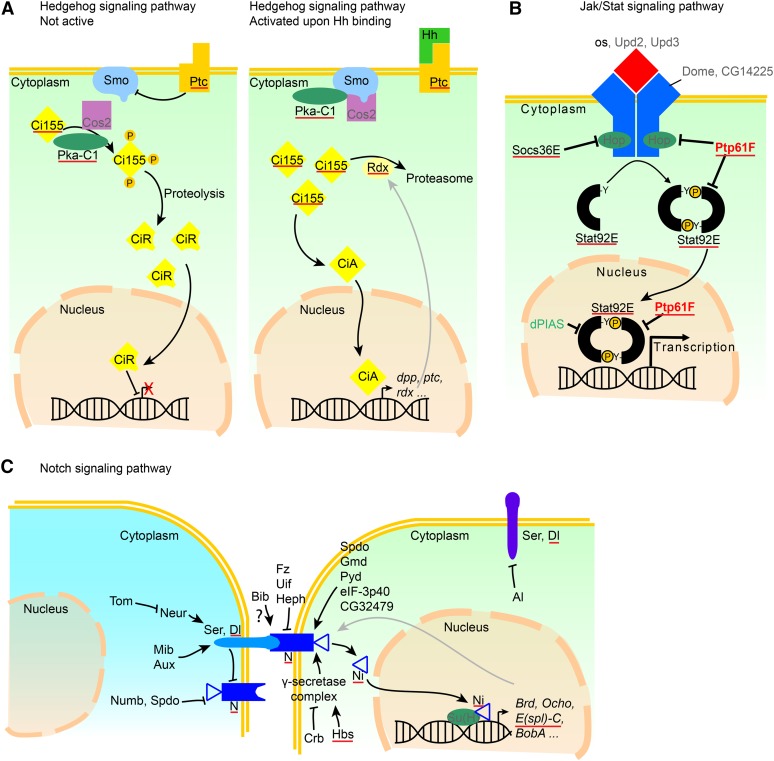
Molecular pathways affected by Gcm. (A) Hedgehog signaling pathway. (B) JAK/STAT signaling pathway. (C) Notch signaling pathway. The proteins and genes in black and red are targeted by Gcm, according to the DamID screen. The genes in red were previously characterized as downstream targets of Gcm, and underlined genes were validated by qPCR in FACS-sorted S2 cells in this study. The proteins in gray are part of the pathway but are not targeted by Gcm.

The Hh pathway and Gcm are necessary for tendon cell differentiation as well as for lamina neuron proliferation and differentiation, but the relation between Gcm and the Hh pathway remained vague (Chotard *et al.* 2005; Umetsu *et al.* 2006). Validation of the DamID screen in cells and *in vivo* reveals that Gcm can control five members of the Hh pathway (basically only *Cos2* was not identified in the screen) (Figure 10A) at tendon cells. This includes *Hh* itself; its receptor, *Ptc*; the proteins transducing the signal *Smo*, *Pka-C1*, and *Ci*; and inhibitor of the signaling pathway Rdx. Interestingly, Chotard *et al.* (2005) proposed that Gcm interacts with the Hh pathway. Together with the finding that the Hh pathway is also required upstream of Gcm in tendon cells for definition of their precursor (Soustelle *et al.* 2004), these data suggest the presence of a feedback loop.

The JAK/STAT signaling pathway triggers lamellocyte differentiation [reviewed in Agaisse and Perrimon (2004) and Myllymaki and Ramet (2014)] and was shown previously to be repressed by Gcm (Jacques *et al.* 2009). The DamID screen shows the direct interaction between Gcm and two major inhibitors of the JAK/STAT pathway, Ptp61F and Socs36E, providing a molecular explanation for the observed genetic interactions. Interestingly, Gcm also targets Stat92E and Os **(**basically all the major players of the JAK/STAT pathway were identified in the screen) (Figure 10B), and the JAK/STAT pathway was shown to induce *gcm* expression in the optic lobe (Wang *et al.* 2013). This suggests the existence of a feedback loop in this cascade as well.

Finally, the N pathway was described as an activator or inhibitor of Gcm in glial cell differentiation depending on the context (Udolph *et al.* 2001; Van de Bor and Giangrande 2001; Umesono *et al.* 2002). The DamID screen shows that Gcm interacts with 30 genes of the N pathway, including seven inhibitors and seven activators of that pathway (Table S1); all the genes in the N pathway were found the in screen (Figure 10C).

Thus, the DamID screen helps to clarify the impact of Gcm on regulatory pathways at the molecular level and paves the way for future studies assessing the biological relevance of these interactions *in vivo*. These pathways had not been characterized previously as regulated by Gcm, nor had they been identified by the three transcriptome assays (Egger *et al.* 2002; Freeman *et al.* 2003; Altenhein *et al.* 2006). This is most likely due to the fact that these pathways involve feedback loops and/or are required in most, if not all, tissues during embryogenesis. Future studies will assess whether Gcm targets different members of a signaling pathway in specific tissues.

### Gcm regulates genes organized in cluster

Analysis of the DamID screen reveals the regulation of genes organized in a cluster, as illustrated by *E(spl)-C*. This observation is in line with the hypothesis that chromatin conformation plays an important role in transcription factor–mediated induction of gene expression. Over a region of 0.8 Mb, the DamID peaks are exclusively concentrated in a region of 50 kb that contains the 12 members of *E(spl)-C* (Figure 7B), whose expression is upregulated by Gcm. In contrast, the unrelated *gro* gene is located at the boundary of the complex, is not controlled by Gcm, and does not contain a DamID peak (Figure 7C). The precise targeting of Gcm to genes within the folded region of *E(spl)-C* correlates well with chromosome conformation capture (3C) analyses, which revealed long-range interactions within the 50-kb complex but very few interactions with regions surrounding it (Schaaf *et al.* 2013). Further development of 3C technology will allow full comprehension of the impact of the chromatin three-dimensional structure on gene expression.

### Gcm regulates genes required for the final function of differentiating tissue

Gcm is generally described as a cell fate determinant. Accordingly, many Gcm direct targets are involved in the early differentiation step, *e.g.*, implementation of glial fate at the expense of neuronal fate in the nervous system (Figure 7A and Figure 8A, pink-shaded area). Surprisingly, a number of genes identified by the screen are necessary at late developmental stages or for function of fully differentiated cells (Figure 7A and Figure 8A, green-shaded area). Typical examples are provided by the genes expressed specifically in the BBB involved in amino acid, sugar, and water exchange and by those coding for septate junction proteins, which are necessary for the filtering function of the BBB (Deligiannaki *et al.* 2015) [reviewed in Hindle and Bainton (2014)]. Similarly, a number of genes acting in the immune system are involved in antigen-specific immune response and the encapsulation of foreign targets. Thus, an early and transiently expressed gene directly targets genes involved in physiologic responses. The fact is that early genes play a broader role that initially was thought not to be so uncommon because this was also observed for the transcription factor Pros in the nervous system. Pros promotes the switch from self-renewal to differentiation. DamID and transcriptome analyses of the downstream targets of Pros revealed that in addition to promoting genes involved in repression of stem cell self-renewal, it also promotes expression of genes involved later on in terminally differentiated neurons (Choksi *et al.* 2006).

### Gcm downstream targets are conserved in mammals

The use of simple organisms such as *Drosophila* to understand the GCM regulatory network in vertebrates has been limited to few studies (Iwasaki *et al.* 2003; Soustelle and Giangrande 2007). This was mostly due to the known requirement of mammalian Gcm genes in placenta or parathyroid glands, two tissues that have no fly equivalent. Despite the disparity of tissues in which Gcm genes are expressed, we were able to find conservation in the target genes. Because in humans *GCM2* downregulation and mutations are associated with parathyroid adenomas and hypoparathyroidism, respectively (Mannstadt *et al.* 2008; Doyle *et al.* 2012; Yi *et al.* 2012; Park *et al.* 2013; Mitsui *et al.* 2014), while *GCM1* downregulation is associated with preeclampsia (Chen *et al.* 2004), the downstream genes identified in this work, including *TBX1*, *GATA* factors, and *FGFR*, represent interesting candidates to dissect the molecular mechanisms underlying these pathologies. Typically, *TBX1* mutations in humans result in DiGeorge syndrome, which includes parathyroid aplasia (Jerome and Papaioannou 2001; Merscher *et al.* 2001). Also, *GATA3* regulates *GCM2* in the parathyroid gland (Grigorieva *et al.* 2010), and *GATA3*, *GATA4*, and *GATA6* are required during trophoblast development [reviewed by Bai *et al.* (2013)]. Our data suggest a feedback loop between *GCM2* and *GATA3* in the parathyroid and point to a hitherto unknown role of *GATA6* in this tissue, in line with recent immunolabeling data (Uhlen *et al.* 2015). Finally, *FGFR1* is misregulated in hyperparathyroidism (Komaba *et al.* 2010).

Finally, the impact of Gcm on the N pathway seems to be conserved to the largest extent. Of the 30 genes identified by the screen, we confirmed 9 by qPCR, including *N*, *E(spl)-C*, and *Dl*. In humans, we show that DLL1 is regulated by GCM1, and in the mouse, the *E(spl)-C* ortholog *Hes5* was reported to be regulated by mGcm1 and mGcm2 (Hitoshi *et al.* 2011). This gives strong support to the hypothesis that Gcm regulates the N pathway in both *Drosophila* and mammals. However, the effect of Gcm most likely depends on the tissue. Indeed, opposite outcomes have already been observed for the effect of Notch on Gcm in *Drosophila* (Udolph *et al.* 2001; Van de Bor and Giangrande 2001; Umesono *et al.* 2002), and both activators and inhibitors of the N pathway were identified in the DamID screen (Table S1). This means that the interaction between Gcm and the N pathway will need to be studied case by case. Overall, our data indicate that even though the main sites of *gcm* expression may be different in mammals and *Drosophila*, the Gcm cascade is at least partially conserved. In this study, we discovered 980 new potential direct targets of Gcm, demonstrated the direct interaction for 36 of them, and the use of *Drosophila* allowed us to discover eight new targets of the GCMs in humans, which include the characterization of a feedback loop for GCMs on themselves.

## References

[bib1] Adolfsen B. Saraswati S. Yoshihara M. Littleton J. T., 2004 Synaptotagmins are trafficked to distinct subcellular domains including the postsynaptic compartment. J. Cell Biol. 166: 249–260. 15263020 10.1083/jcb.200312054 PMC2172321

[bib2] Agaisse H. Perrimon N., 2004 The roles of JAK/STAT signaling in Drosophila immune responses. Immunol. Rev. 198: 72–82. 15199955 10.1111/j.0105-2896.2004.0133.x

[bib3] Akiyama Y. Hosoya T. Poole A. M. Hotta Y., 1996 The gcm-motif: a novel DNA-binding motif conserved in Drosophila and mammals. Proc. Natl. Acad. Sci. USA 93: 14912–14916. 8962155 10.1073/pnas.93.25.14912 PMC26236

[bib4] Al-Anzi B. Wyman R. J., 2009 The Drosophila immunoglobulin gene turtle encodes guidance molecules involved in axon pathfinding. Neural Dev. 4: 31. 19686588 10.1186/1749-8104-4-31 PMC2739522

[bib5] Alfonso T. B. Jones B. W., 2002 gcm2 promotes glial cell differentiation and is required with glial cells missing for macrophage development in Drosophila. Dev. Biol. 248: 369–383. 12167411 10.1006/dbio.2002.0740

[bib6] Altenhein B. Becker A. Busold C. Beckmann B. Hoheisel J. D., 2006 Expression profiling of glial genes during Drosophila embryogenesis. Dev. Biol. 296: 545–560. 16762338 10.1016/j.ydbio.2006.04.460

[bib7] Anson-Cartwright L. Dawson K. Holmyard D. Fisher S. J. Lazzarini R. A., 2000 The glial cells missing-1 protein is essential for branching morphogenesis in the chorioallantoic placenta. Nat. Genet. 25: 311–314. 10888880 10.1038/77076

[bib8] Anteby E. Y. Natanson-Yaron S. Hamani Y. Sciaki Y. Goldman-Wohl D., 2005 Fibroblast growth factor-10 and fibroblast growth factor receptors 1–4: expression and peptide localization in human decidua and placenta. Eur. J. Obstet. Gynecol. Reprod. Biol. 119: 27–35. 15734081 10.1016/j.ejogrb.2004.05.014

[bib9] Armitage S. A. Freiburg R. Y. Kurtz J. Bravo I. G., 2012 The evolution of Dscam genes across the arthropods. BMC Evol. Biol. 12: 53. 22500922 10.1186/1471-2148-12-53 PMC3364881

[bib10] Artavanis-Tsakonas S. Rand M. D. Lake R. J., 1999 Notch signaling: Cell fate control and signal integration in development. Science 284: 770–776. 10221902 10.1126/science.284.5415.770

[bib11] Avadhanula V. Weasner B. P. Hardy G. G. Kumar J. P. Hardy R. W., 2009 A novel system for the launch of alphavirus RNA synthesis reveals a role for the Imd pathway in arthropod antiviral response. PLoS Pathog. 5: e1000582. 10.1371/journal.ppat.1000582 PMC2738967 19763182

[bib12] Avet-Rochex A. Perrin J. Bergeret E. Fauvarque M. O., 2007 Rac2 is a major actor of Drosophila resistance to Pseudomonas aeruginosa acting in phagocytic cells. Genes Cells 12: 1193–1204. 17903178 10.1111/j.1365-2443.2007.01121.x

[bib13] Awasaki T. Lai S. L. Ito K. Lee T., 2008 Organization and postembryonic development of glial cells in the adult central brain of Drosophila. J. Neurosci. 28: 13742–13753. 19091965 10.1523/JNEUROSCI.4844-08.2008 PMC6671902

[bib14] Baczyk D. Drewlo S. Proctor L. Dunk C. Lye S., 2009 Glial cell missing-1 transcription factor is required for the differentiation of the human trophoblast. Cell Death Differ. 16: 719–727. 19219068 10.1038/cdd.2009.1

[bib15] Baeg G. H. Zhou R. Perrimon N., 2005 Genome-wide RNAi analysis of JAK/STAT signaling components in Drosophila. Genes Dev. 19: 1861–1870. 16055650 10.1101/gad.1320705 PMC1186186

[bib16] Bahri S. M. Chia W. Yang X. H., 2001 Characterization and mutant analysis of the Drosophila sema 5C gene. Dev. Dyn. 221: 322–330. 11458392 10.1002/dvdy.1142

[bib17] Bai H. Sakurai T. Godkin J. D. Imakawa K., 2013 Expression and potential role of GATA factors in trophoblast development. J. Reprod. Dev. 59: 1–6. 23428586 10.1262/jrd.2012-100 PMC3943230

[bib18] Bailey A. M. Posakony J. W., 1995 Suppressor of hairless directly activates transcription of enhancer of split complex genes in response to Notch receptor activity. Genes Dev. 9: 2609–2622. 7590239 10.1101/gad.9.21.2609

[bib19] Basyuk E. Cross J. C. Corbin J. Nakayama H. Hunter P., 1999 Murine Gcm1 gene is expressed in a subset of placental trophoblast cells. Dev. Dyn. 214: 303–311. 10213386 10.1002/(SICI)1097-0177(199904)214:4<303::AID-AJA3>3.0.CO;2-B

[bib20] Bates C. M., 2011 Role of fibroblast growth factor receptor signaling in kidney development. Pediatr. Nephrol. 26: 1373–1379. 21222001 10.1007/s00467-010-1747-z PMC4007488

[bib21] Berger C. Pallavi S. K. Prasad M. Shashidhara L. S. Technau G. M., 2005 a A critical role for cyclin E in cell fate determination in the central nervous system of Drosophila melanogaster. Nat. Cell Biol. 7: 56–62. 15580266 10.1038/ncb1203

[bib22] Berger C. Pallavi S. K. Prasad M. Shashidhara L. S. Technau G. M., 2005 b Cyclin E acts under the control of Hox-genes as a cell fate determinant in the developing central nervous system. Cell Cycle 4: 422–425. 15684605 10.4161/cc.4.3.1524

[bib23] Bernardoni R. Vivancos V. Giangrande A., 1997 glide/gcm is expressed and required in the scavenger cell lineage. Dev. Biol. 191: 118–130. 9356176 10.1006/dbio.1997.8702

[bib24] Bernardoni R. Miller A. A. Giangrande A., 1998 Glial differentiation does not require a neural ground state. Development 125: 3189–3200. 9671591 10.1242/dev.125.16.3189

[bib25] Bischof J. Maeda R. K. Hediger M. Karch F. Basler K., 2007 An optimized transgenesis system for Drosophila using germ-line-specific phi C31 integrases. Proc. Natl. Acad. Sci. USA 104: 3312–3317. 17360644 10.1073/pnas.0611511104 PMC1805588

[bib26] Blanchette M. Kent W. J. Riemer C. Elnitski L. Smit A. F., 2004 Aligning multiple genomic sequences with the threaded blockset aligner. Genome Res. 14: 708–715. 15060014 10.1101/gr.1933104 PMC383317

[bib27] Bottenberg W. Sanchez-Soriano N. Alves-Silva J. Hahn I. Mende M., 2009 Context-specific requirements of functional domains of the Spectraplakin Short stop in vivo. Mech. Dev. 126: 489–502. 19409984 10.1016/j.mod.2009.04.004

[bib28] Callus B. A. Mathey-Prevot B., 2002 SOCS36E, a novel Drosophila SOCS protein, suppresses JAK/STAT and EGF-R signalling in the imaginal wing disc. Oncogene 21: 4812–4821. 12101419 10.1038/sj.onc.1205618

[bib29] Campbell G. Goring H. Lin T. Spana E. Andersson S., 1994 RK2, a glial-specific homeodomain protein required for embryonic nerve cord condensation and viability in Drosophila. Development 120: 2957–2966. 7607085 10.1242/dev.120.10.2957

[bib30] Cantor A. B. Orkin S. H., 2005 Coregulation of GATA factors by the Friend of GATA (FOG) family of multitype zinc finger proteins. Semin. Cell Dev. Biol. 16: 117–128. 15659346 10.1016/j.semcdb.2004.10.006

[bib31] Carney T. D. Miller M. R. Robinson K. J. Bayraktar O. A. Osterhout J. A., 2012 Functional genomics identifies neural stem cell sub-type expression profiles and genes regulating neuroblast homeostasis. Dev. Biol. 361: 137–146. 22061480 10.1016/j.ydbio.2011.10.020 PMC4110207

[bib32] Cattenoz P. B. Giangrande A., 2015 New insights in the clockwork mechanism regulating lineage specification: Lessons from the Drosophila nervous system. Dev. Dyn. 244: 332–341. 25399853 10.1002/dvdy.24228

[bib33] Ceron J. Gonzalez C. Tejedor F. J., 2001 Patterns of cell division and expression of asymmetric cell fate determinants in postembryonic neuroblast lineages of Drosophila. Dev. Biol. 230: 125–138. 11161567 10.1006/dbio.2000.0110

[bib34] Charvet B. Ruggiero F. Le Guellec D., 2012 The development of the myotendinous junction: a review. Muscles Ligaments Tendons J. 2: 53–63. 23738275 PMC3666507

[bib35] Chen C. P. Chen C. Y. Yang Y. C. Su T. H. Chen H., 2004 Decreased placental GCM1 (glial cells missing) gene expression in pre-eclampsia. Placenta 25: 413–421. 15081636 10.1016/j.placenta.2003.10.014

[bib36] Chen H. Boutros P. C., 2011 VennDiagram: a package for the generation of highly-customizable Venn and Euler diagrams in R. BMC Bioinformatics 12: 35. 21269502 10.1186/1471-2105-12-35 PMC3041657

[bib37] Chen T. Bunting M. Karim F. D. Thummel C. S., 1992 Isolation and characterization of five Drosophila genes that encode an ets-related DNA binding domain. Dev. Biol. 151: 176–191. 1577186 10.1016/0012-1606(92)90225-6

[bib38] Cherbas L. Willingham A. Zhang D. Yang L. Zou Y., 2011 The transcriptional diversity of 25 Drosophila cell lines. Genome Res. 21: 301–314. 21177962 10.1101/gr.112961.110 PMC3032933

[bib39] Chlon T. M. Crispino J. D., 2012 Combinatorial regulation of tissue specification by GATA and FOG factors. Development 139: 3905–3916. 23048181 10.1242/dev.080440 PMC3472596

[bib40] Cho N. K. Keyes L. Johnson E. Heller J. Ryner L., 2002 Developmental control of blood cell migration by the Drosophila VEGF pathway. Cell 108: 865–876. 11955438 10.1016/s0092-8674(02)00676-1

[bib41] Choksi S. P. Southall T. D. Bossing T. Edoff K. de Wit E., 2006 Prospero acts as a binary switch between self-renewal and differentiation in Drosophila neural stem cells. Dev. Cell 11: 775–789. 17141154 10.1016/j.devcel.2006.09.015

[bib42] Chotard C. Leung W. Salecker I., 2005 Glial cells missing and gcm2 cell autonomously regulate both glial and neuronal development in the visual system of Drosophila. Neuron 48: 237–251. 16242405 10.1016/j.neuron.2005.09.019

[bib43] Claeys I. Simonet G. Poels J. Van Loy T. Vercammen L., 2002 Insulin-related peptides and their conserved signal transduction pathway. Peptides 23: 807–816. 11897402 10.1016/s0196-9781(01)00666-0

[bib44] Correa P. Akerstrom G. Westin G., 2002 Underexpression of Gcm2, a master regulatory gene of parathyroid gland development, in adenomas of primary hyperparathyroidism. Clin. Endocrinol. 57: 501–505. 10.1046/j.1365-2265.2002.01627.x 12354132

[bib45] Cronin S. J. Nehme N. T. Limmer S. Liegeois S. Pospisilik J. A., 2009 Genome-wide RNAi screen identifies genes involved in intestinal pathogenic bacterial infection. Science 325: 340–343. 19520911 10.1126/science.1173164 PMC2975362

[bib46] Daniel A. Dumstrei K. Lengyel J. A. Hartenstein V., 1999 The control of cell fate in the embryonic visual system by atonal, tailless and EGFR signaling. Development 126: 2945–2954. 10357938 10.1242/dev.126.13.2945

[bib47] Dantoft W. Davis M. M. Lindvall J. M. Tang X. Uvell H., 2013 The Oct1 homolog Nubbin is a repressor of NF-kappaB-dependent immune gene expression that increases the tolerance to gut microbiota. BMC Biol. 11: 99. 24010524 10.1186/1741-7007-11-99 PMC3849502

[bib48] De Iaco R. Soustelle L. Kammerer M. Sorrentino S. Jacques C., 2006 Huckebein-mediated autoregulation of Glide/Gcm triggers glia specification. EMBO J. 25: 244–254. 16362045 10.1038/sj.emboj.7600907 PMC1356350

[bib49] Deligiannaki M. Casper A. L. Jung C. Gaul U., 2015 Pasiflora proteins are novel core components of the septate junction. Development 142: 3046–3057. 26329602 10.1242/dev.119412 PMC4582180

[bib50] Desai C. J. Gindhart J. G. Goldstein L. S. B. Zinn K., 1996 Receptor tyrosine phosphatases are required for motor axon guidance in the Drosophila embryo. Cell 84: 599–609. 8598046 10.1016/s0092-8674(00)81035-1

[bib51] DeSalvo M. K. Hindle S. J. Rusan Z. M. Orng S. Eddison M., 2014 The Drosophila surface glia transcriptome: evolutionary conserved blood-brain barrier processes. Front. Neurosci. 8: 346. 25426014 10.3389/fnins.2014.00346 PMC4224204

[bib52] Dijkers P. F. O’Farrell P. H., 2007 Drosophila calcineurin promotes induction of innate immune responses. Curr. Biol. 17: 2087–2093. 18060786 10.1016/j.cub.2007.11.001 PMC2180389

[bib53] dos Santos G. Schroeder A. J. Goodman J. L. Strelets V. B. Crosby M. A., 2015 FlyBase: introduction of the Drosophila melanogaster release 6 reference genome assembly and large-scale migration of genome annotations. Nucleic Acids Res. 43: D690–D697. 25398896 10.1093/nar/gku1099 PMC4383921

[bib54] Doyle D. Kirwin S. M. Sol-Church K. Levine M. A., 2012 A novel mutation in the GCM2 gene causing severe congenital isolated hypoparathyroidism. J. Pediatr. Endocrinol. Metab. 25: 741–746. 23155703 10.1515/jpem-2012-0080 PMC3694175

[bib55] Dworak H. A. Charles M. A. Pellerano L. B. Sink H., 2001 Characterization of Drosophila hibris, a gene related to human nephrin. Development 128: 4265–4276. 11684662 10.1242/dev.128.21.4265

[bib56] Edenfeld G. Altenhein B. Zierau A. Cleppien D. Krukkert K., 2007 Notch and Numb are required for normal migration of peripheral glia in Drosophila. Dev. Biol. 301: 27–37. 17157832 10.1016/j.ydbio.2006.11.013

[bib57] Egger B. Leemans R. Loop T. Kammermeier L. Fan Y., 2002 Gliogenesis in Drosophila: genome-wide analysis of downstream genes of glial cells missing in the embryonic nervous system. Development 129: 3295–3309. 12091301 10.1242/dev.129.14.3295

[bib58] Egger B. Gold K. S. Brand A. H., 2010 Notch regulates the switch from symmetric to asymmetric neural stem cell division in the Drosophila optic lobe. Development 137: 2981–2987. 20685734 10.1242/dev.051250 PMC2926952

[bib59] Engel E. Viargues P. Mortier M. Taillebourg E. Coute Y., 2014 Identifying USPs regulating immune signals in Drosophila: USP2 deubiquitinates Imd and promotes its degradation by interacting with the proteasome. Cell Commun. Signal. 12: 41. 25027767 10.1186/s12964-014-0041-2 PMC4140012

[bib60] Evans C. J. Banerjee U., 2003 Transcriptional regulation of hematopoiesis in Drosophila. Blood Cells Mol. Dis. 30: 223–228. 12732186 10.1016/s1079-9796(03)00028-7

[bib61] Flici H. Cattenoz P. B. Komonyi O. Laneve P. Erkosar B., 2014 Interlocked loops trigger lineage specification and stable fates in the Drosophila nervous system. Nat. Commun. 5: 4484. 25066644 10.1038/ncomms5484

[bib62] Fossett N. Tevosian S. G. Gajewski K. Zhang Q. Orkin S. H., 2001 The Friend of GATA proteins U-shaped, FOG-1, and FOG-2 function as negative regulators of blood, heart, and eye development in Drosophila. Proc. Natl. Acad. Sci. USA 98: 7342–7347. 11404479 10.1073/pnas.131215798 PMC34670

[bib63] Franc N. C. Heitzler P. Ezekowitz R. A. White K., 1999 Requirement for croquemort in phagocytosis of apoptotic cells in Drosophila. Science 284: 1991–1994. 10373118 10.1126/science.284.5422.1991

[bib64] Frandsen J. L. Gunn B. Muratoglu S. Fossett N. Newfeld S. J., 2008 Salmonella pathogenesis reveals that BMP signaling regulates blood cell homeostasis and immune responses in Drosophila. Proc. Natl. Acad. Sci. USA 105: 14952–14957. 18815369 10.1073/pnas.0808208105 PMC2553038

[bib65] Franzdottir S. R. Engelen D. Yuva-Aydemir Y. Schmidt I. Aho A., 2009 Switch in FGF signalling initiates glial differentiation in the Drosophila eye. Nature 460: 758–761. 19597479 10.1038/nature08167

[bib66] Freeman M. R. Doe C. Q., 2001 Asymmetric Prospero localization is required to generate mixed neuronal/glial lineages in the Drosophila CNS. Development 128: 4103–4112. 11641232 10.1242/dev.128.20.4103

[bib67] Freeman M. R. Delrow J. Kim J. Johnson E. Doe C. Q., 2003 Unwrapping glial biology: Gcm target genes regulating glial development, diversification, and function. Neuron 38: 567–580. 12765609 10.1016/s0896-6273(03)00289-7

[bib68] Garbe J. C. Yang E. Fristrom J. W., 1993 IMP-L2: an essential secreted immunoglobulin family member implicated in neural and ectodermal development in Drosophila. Development 119: 1237–1250. 8306886 10.1242/dev.119.4.1237

[bib69] Gasperowicz M. Rai A. Cross J. C., 2013 Spatiotemporal expression of Notch receptors and ligands in developing mouse placenta. Gene Expr. Patterns 13: 249–254. 23665443 10.1016/j.gep.2013.04.006

[bib70] Ghazi A. Paul L. VijayRaghavan K., 2003 Prepattern genes and signaling molecules regulate stripe expression to specify Drosophila flight muscle attachment sites. Mech. Dev. 120: 519–528. 12782269 10.1016/s0925-4773(03)00042-x

[bib71] Giesen K. Hummel T. Stollewerk A. Harrison S. Travers A., 1997 Glial development in the Drosophila CNS requires concomitant activation of glial and repression of neuronal differentiation genes. Development 124: 2307–2316. 9199357 10.1242/dev.124.12.2307

[bib72] Goldstein L. S. B. Gunawardena S., 2000 Flying through the Drosophila cytoskeletal genome. J. Cell Biol. 150: F63–F68. 10908588 10.1083/jcb.150.2.f63 PMC2180230

[bib73] Gonzalez F. Romani S. Cubas P. Modolell J. Campuzano S., 1989 Molecular analysis of the asense gene, a member of the achaete-scute complex of Drosophila melanogaster, and its novel role in optic lobe development. EMBO J. 8: 3553–3562. 2510998 10.1002/j.1460-2075.1989.tb08527.x PMC402034

[bib74] Gordon J. Bennett A. R. Blackburn C. C. Manley N. R., 2001 Gcm2 and Foxn1 mark early parathyroid- and thymus-specific domains in the developing third pharyngeal pouch. Mech. Dev. 103: 141–143. 11335122 10.1016/s0925-4773(01)00333-1

[bib75] Gordon M. D. Dionne M. S. Schneider D. S. Nusse R., 2005 WntD is a feedback inhibitor of dorsal/NF-κB in Drosophila development and immunity. Nature 437: 746–749. 16107793 10.1038/nature04073 PMC1256032

[bib76] Gorski S. M. Chittaranjan S. Pleasance E. D. Freeman J. D. Anderson C. L., 2003 A SAGE approach to discovery of genes involved in autophagic cell death. Curr. Biol. 13: 358–363. 12593804 10.1016/s0960-9822(03)00082-4

[bib77] Granderath S. Stollewerk A. Greig S. Goodman C. S. O’Kane C. J., 1999 loco encodes an RGS protein required for Drosophila glial differentiation. Development 126: 1781–1791. 10079238 10.1242/dev.126.8.1781

[bib78] Granderath S. Bunse I. Klambt C., 2000 Gcm and pointed synergistically control glial transcription of the Drosophila gene loco. Mech. Dev. 91: 197–208. 10704844 10.1016/s0925-4773(99)00304-4

[bib79] Graveley B. R. Brooks A. N. Carlson J. W. Duff M. O. Landolin J. M., 2011 The developmental transcriptome of Drosophila melanogaster. Nature 471: 473–479. 21179090 10.1038/nature09715 PMC3075879

[bib80] Griffiths R. L. Hidalgo A., 2004 Prospero maintains the mitotic potential of glial precursors enabling them to respond to neurons. EMBO J. 23: 2440–2450. 15167898 10.1038/sj.emboj.7600258 PMC423295

[bib81] Grigorieva I. V. Mirczuk S. Gaynor K. U. Nesbit M. A. Grigorieva E. F., 2010 Gata3-deficient mice develop parathyroid abnormalities due to dysregulation of the parathyroid-specific transcription factor Gcm2. J. Clin. Invest. 120: 2144–2155. 20484821 10.1172/JCI42021 PMC2877956

[bib254] Guillermin O. Perruchoud B. Sprecher S. G. Egger B., 2015 Characterization of tailless functions during Drosophila optic lobe formation. Developmental Biology 405: 202–213. 26111972 10.1016/j.ydbio.2015.06.011

[bib82] Guntermann S. Primrose D. A. Foley E., 2009 Dnr1-dependent regulation of the Drosophila immune deficiency signaling pathway. Dev. Comp. Immunol. 33: 127–134. 18775745 10.1016/j.dci.2008.07.021

[bib83] Gunther T. Chen Z. F. Kim J. S. Priemel M. Rueger J. M., 2000 Genetic ablation of parathyroid glands reveals another source of parathyroid hormone. Nature 406: 199–203. 10910362 10.1038/35018111

[bib84] Hadzic T. Park D. Abruzzi K. C. Yang L. Trigg J. S., 2015 Genome-wide features of neuroendocrine regulation in Drosophila by the basic helix-loop-helix transcription factor DIMMED. Nucleic Acids Res. 43: 2199–2215. 25634895 10.1093/nar/gku1377 PMC4344488

[bib85] Halter D. A. Urban J. Rickert C. Ner S. S. Ito K., 1995 The homeobox gene repo is required for the differentiation and maintenance of glia function in the embryonic nervous system of Drosophila melanogaster. Development 121: 317–332. 7768175 10.1242/dev.121.2.317

[bib86] Hammonds A. S. Bristow C. A. Fisher W. W. Weiszmann R. Wu S., 2013 Spatial expression of transcription factors in Drosophila embryonic organ development. Genome Biol. 14: R140. 24359758 10.1186/gb-2013-14-12-r140 PMC4053779

[bib87] Han Z. Olson E. N., 2005 Hand is a direct target of Tinman and GATA factors during Drosophila cardiogenesis and hematopoiesis. Development 132: 3525–3536. 15975941 10.1242/dev.01899

[bib88] Hatini V. DiNardo S., 2001 Distinct signals generate repeating striped pattern in the embryonic parasegment. Mol. Cell 7: 151–160. 11172720 10.1016/s1097-2765(01)00163-0

[bib89] Herr F. Schreiner I. Baal N. Pfarrer C. Zygmunt M., 2011 Expression patterns of Notch receptors and their ligands Jagged and Delta in human placenta. Placenta 32: 554–563. 21726900 10.1016/j.placenta.2011.04.018

[bib90] Hewes R. S. Park D. Gauthier S. A. Schaefer A. M. Taghert P. H., 2003 The bHLH protein Dimmed controls neuroendocrine cell differentiation in Drosophila. Development 130: 1771–1781. 12642483 10.1242/dev.00404

[bib91] Hidalgo A. Kinrade E. F. Georgiou M., 2001 The Drosophila neuregulin vein maintains glial survival during axon guidance in the CNS. Dev. Cell 1: 679–690. 11709188 10.1016/s1534-5807(01)00074-0

[bib92] Hindle S. J. Bainton R. J., 2014 Barrier mechanisms in the Drosophila blood-brain barrier. Front. Neurosci. 8: 414. 25565944 10.3389/fnins.2014.00414 PMC4267209

[bib93] Hiramoto M. Hiromi Y., 2006 ROBO directs axon crossing of segmental boundaries by suppressing responsiveness to relocalized Netrin. Nat. Neurosci. 9: 58–66. 16341212 10.1038/nn1612

[bib94] Hitoshi S. Ishino Y. Kumar A. Jasmine S. Tanaka K. F., 2011 Mammalian Gcm genes induce Hes5 expression by active DNA demethylation and induce neural stem cells. Nat. Neurosci. 14: 957–964. 21765423 10.1038/nn.2875

[bib95] Hofmeyer K. Treisman J. E., 2009 The receptor protein tyrosine phosphatase LAR promotes R7 photoreceptor axon targeting by a phosphatase-independent signaling mechanism. Proc. Natl. Acad. Sci. USA 106: 19399–19404. 19889974 10.1073/pnas.0903961106 PMC2780745

[bib96] Hosoya T. Takizawa K. Nitta K. Hotta Y., 1995 Glial cells missing: a binary switch between neuronal and glial determination in Drosophila. Cell 82: 1025–1036. 7553844 10.1016/0092-8674(95)90281-3

[bib97] Hou Y. C. Chittaranjan S. Barbosa S. G. McCall K. Gorski S. M., 2008 Effector caspase Dcp-1 and IAP protein Bruce regulate starvation-induced autophagy during Drosophila melanogaster oogenesis. J. Cell Biol. 182: 1127–1139. 18794330 10.1083/jcb.200712091 PMC2542474

[bib98] Howell L. Sampson C. J. Xavier M. J. Bolukbasi E. Heck M. M., 2012 A directed miniscreen for genes involved in the Drosophila anti-parasitoid immune response. Immunogenetics 64: 155–161. 21947570 10.1007/s00251-011-0571-3

[bib99] Hu Y. Flockhart I. Vinayagam A. Bergwitz C. Berger B., 2011 An integrative approach to ortholog prediction for disease-focused and other functional studies. BMC Bioinformatics 12: 357. 21880147 10.1186/1471-2105-12-357 PMC3179972

[bib100] Huang da W. Sherman B. T. Lempicki R. A., 2009 a Bioinformatics enrichment tools: paths toward the comprehensive functional analysis of large gene lists. Nucleic Acids Res. 37: 1–13. 19033363 10.1093/nar/gkn923 PMC2615629

[bib101] Huang da W. Sherman B. T. Lempicki R. A., 2009 b Systematic and integrative analysis of large gene lists using DAVID bioinformatics resources. Nat. Protoc. 4: 44–57. 19131956 10.1038/nprot.2008.211

[bib102] Huang Z. Kunes S., 1996 Hedgehog, transmitted along retinal axons, triggers neurogenesis in the developing visual centers of the Drosophila brain. Cell 86: 411–422. 8756723 10.1016/s0092-8674(00)80114-2

[bib103] Huang Z. Kunes S., 1998 Signals transmitted along retinal axons in Drosophila: Hedgehog signal reception and the cell circuitry of lamina cartridge assembly. Development 125: 3753–3764. 9729484 10.1242/dev.125.19.3753

[bib104] Iwasaki Y. Hosoya T. Takebayashi H. Ogawa Y. Hotta Y., 2003 The potential to induce glial differentiation is conserved between Drosophila and mammalian glial cells missing genes. Development 130: 6027–6035. 14573516 10.1242/dev.00822

[bib105] Jacques C. Soustelle L. Nagy I. Diebold C. Giangrande A., 2009 A novel role of the glial fate determinant glial cells missing in hematopoiesis. Int. J. Dev. Biol. 53: 1013–1022. 19598118 10.1387/ijdb.082726cj

[bib106] Jerome L. A. Papaioannou V. E., 2001 DiGeorge syndrome phenotype in mice mutant for the T-box gene, Tbx1. Nat. Genet. 27: 286–291. 11242110 10.1038/85845

[bib107] Jhaveri D. Saharan S. Sen A. Rodrigues V., 2004 Positioning sensory terminals in the olfactory lobe of Drosophila by Robo signaling. Development 131: 1903–1912. 15056612 10.1242/dev.01083

[bib108] Ji S. Sun M. Zheng X. Li L. Sun L., 2014 Cell-surface localization of Pellino antagonizes Toll-mediated innate immune signalling by controlling MyD88 turnover in Drosophila. Nat. Commun. 5: 3458. 24632597 10.1038/ncomms4458 PMC3959197

[bib109] Jin L. H. Shim J. Yoon J. S. Kim B. Kim J., 2008 Identification and functional analysis of antifungal immune response genes in Drosophila. PLoS Pathog. 4: e1000168. 10.1371/journal.ppat.1000168 PMC2542415 18833296

[bib110] Jones B. W. Fetter R. D. Tear G. Goodman C. S., 1995 Glial cells missing: a genetic switch that controls glial *vs.* neuronal fate. Cell 82: 1013–1023. 7553843 10.1016/0092-8674(95)90280-5

[bib111] Kammerer M. Giangrande A., 2001 Glide2, a second glial promoting factor in Drosophila melanogaster. EMBO J. 20: 4664–4673. 11532931 10.1093/emboj/20.17.4664 PMC125586

[bib112] Karlsson C. Korayem A. M. Scherfer C. Loseva O. Dushay M. S., 2004 Proteomic analysis of the Drosophila larval hemolymph clot. J. Biol. Chem. 279: 52033–52041. 15466469 10.1074/jbc.M408220200

[bib113] Karlstrom R. O. Wilder L. P. Bastiani M. J., 1993 Lachesin: an immunoglobulin superfamily protein whose expression correlates with neurogenesis in grasshopper embryos. Development 118: 509–522. 8223276 10.1242/dev.118.2.509

[bib114] Katoh M. Nakagama H., 2014 FGF receptors: cancer biology and therapeutics. Med. Res. Rev. 34: 280–300. 23696246 10.1002/med.21288

[bib115] Kawamori H. Tai M. Sato M. Yasugi T. Tabata T., 2011 Fat/Hippo pathway regulates the progress of neural differentiation signaling in the Drosophila optic lobe. Dev. Growth Differ. 53: 653–667. 21671914 10.1111/j.1440-169X.2011.01279.x

[bib116] Kelleher F. C. O’Sullivan H. Smyth E. McDermott R. Viterbo A., 2013 Fibroblast growth factor receptors, developmental corruption and malignant disease. Carcinogenesis 34: 2198–2205. 23880303 10.1093/carcin/bgt254

[bib117] Kim J. Jones B. W. Zock C. Chen Z. Wang H., 1998 Isolation and characterization of mammalian homologs of the Drosophila gene glial cells missing. Proc. Natl. Acad. Sci. USA 95: 12364–12369. 9770492 10.1073/pnas.95.21.12364 PMC22837

[bib118] Kim J. H. Wang X. Coolon R. Ye B., 2013 Dscam expression levels determine presynaptic arbor sizes in Drosophila sensory neurons. Neuron 78: 827–838. 23764288 10.1016/j.neuron.2013.05.020 PMC3709448

[bib119] Kim L. K. Choi U. Y. Cho H. S. Lee J. S. Lee W. B., 2007 Down-regulation of NF-κB target genes by the AP-1 and STAT complex during the innate immune response in Drosophila. PLoS Biol. 5: e238. 10.1371/journal.pbio.0050238 PMC1964775 17803358

[bib120] Klambt C., 1993 The Drosophila gene pointed encodes two ETS-like proteins which are involved in the development of the midline glial cells. Development 117: 163–176. 8223245 10.1242/dev.117.1.163

[bib121] Klambt C. Glazer L. Shilo B. Z., 1992 Breathless, a Drosophila FGF receptor homolog, is essential for migration of tracheal and specific midline glial cells. Genes Dev. 6: 1668–1678. 1325393 10.1101/gad.6.9.1668

[bib122] Kleino A. Valanne S. Ulvila J. Kallio J. Myllymaki H., 2005 Inhibitor of apoptosis 2 and TAK1-binding protein are components of the Drosophila Imd pathway. EMBO J. 24: 3423–3434. 16163390 10.1038/sj.emboj.7600807 PMC1276168

[bib123] Komaba H. Goto S. Fujii H. Hamada Y. Kobayashi A., 2010 Depressed expression of Klotho and FGF receptor 1 in hyperplastic parathyroid glands from uremic patients. Kidney Int. 77: 232–238. 19890272 10.1038/ki.2009.414

[bib124] Konrad L. Becker G. Schmidt A. Klockner T. Kaufer-Stillger G., 1994 Cloning, structure, cellular localization, and possible function of the tumor suppressor gene lethal(3)malignant blood neoplasm-1 of Drosophila melanogaster. Dev. Biol. 163: 98–111. 8174791 10.1006/dbio.1994.1126

[bib125] Kramer S. G. Kidd T. Simpson J. H. Goodman C. S., 2001 Switching repulsion to attraction: changing responses to slit during transition in mesoderm migration. Science 292: 737–740. 11326102 10.1126/science.1058766

[bib126] Krasnow M. A. Saffman E. E. Kornfeld K. Hogness D. S., 1989 Transcriptional activation and repression by ultrabithorax proteins in cultured Drosophila cells. Cell 57: 1031–1043. 2567632 10.1016/0092-8674(89)90341-3

[bib127] Krueger N. X. VanVactor D. Wan H. I. Gelbart W. M. Goodman C. S., 1996 The transmembrane tyrosine phosphatase DLAR controls motor axon guidance in Drosophila. Cell 84: 611–622. 8598047 10.1016/s0092-8674(00)81036-3

[bib128] Kumar A. Gupta T. Berzsenyi S. Giangrande A., 2015 N-cadherin negatively regulates collective Drosophila glial migration through actin cytoskeleton remodeling. J. Cell Sci. 128: 900–912. 25593128 10.1242/jcs.157974

[bib129] Kussel P. Frasch M., 1995 Pendulin, a Drosophila protein with cell cycle-dependent nuclear-localization, is required for normal-cell proliferation. J. Cell Biol. 129: 1491–1507. 7790350 10.1083/jcb.129.6.1491 PMC2291176

[bib130] Lahaye L. L. Wouda R. R. de Jong A. W. M. Fradkin L. G. Noordermeer J. N., 2012 WNT5 Interacts with the Ryk receptors doughnut and derailed to mediate muscle attachment site selection in Drosophila melanogaster. PLoS One 7: e32297. 10.1371/journal.pone.0032297 PMC3293800 22403643

[bib131] Laneve P. Delaporte C. Trebuchet G. Komonyi O. Flici H., 2013 The Gcm/Glide molecular and cellular pathway: new actors and new lineages. Dev. Biol. 375: 65–78. 23276603 10.1016/j.ydbio.2012.12.014

[bib132] Leal S. M. Qian L. Lacin H. Bodmer R. Skeath J. B., 2009 Neuromancer1 and Neuromancer2 regulate cell fate specification in the developing embryonic CNS of Drosophila melanogaster. Dev. Biol. 325: 138–150. 19013145 10.1016/j.ydbio.2008.10.006 PMC2648533

[bib133] Lebestky T. Chang T. Hartenstein V. Banerjee U., 2000 Specification of Drosophila hematopoietic lineage by conserved transcription factors. Science 288: 146–149. 10753120 10.1126/science.288.5463.146

[bib134] Lebestky T. Jung S. H. Banerjee U., 2003 A Serrate-expressing signaling center controls Drosophila hematopoiesis. Genes Dev. 17: 348–353. 12569125 10.1101/gad.1052803 PMC195988

[bib135] Lee B. P. Jones B. W., 2005 Transcriptional regulation of the Drosophila glial gene repo. Mech. Dev. 122: 849–862. 15939231 10.1016/j.mod.2005.01.002

[bib136] Lekven A. C. Tepass U. Keshmeshian M. Hartenstein V., 1998 Faint sausage encodes a novel extracellular protein of the immunoglobulin superfamily required for cell migration and the establishment of normal axonal pathways in the Drosophila nervous system. Development 125: 2747–2758. 9636088 10.1242/dev.125.14.2747

[bib137] Limmer S. Weiler A. Volkenhoff A. Babatz F. Klambt C., 2014 The Drosophila blood-brain barrier: development and function of a glial endothelium. Front. Neurosci. 8: 365. 25452710 10.3389/fnins.2014.00365 PMC4231875

[bib138] Lin W. H. Huang L. H. Yeh J. Y. Hoheisel J. Lehrach H., 1995 Expression of a Drosophila GATA transcription factor in multiple tissues in the developing embryos. Identification of homozygous lethal mutants with P-element insertion at the promoter region. J. Biol. Chem. 270: 25150–25158. 7559649 10.1074/jbc.270.42.25150

[bib139] Loer B. Bauer R. Bornheim R. Grell J. Kremmer E., 2008 The NHL-domain protein Wech is crucial for the integrin-cytoskeleton link. Nat. Cell Biol. 10: 422–428. 18327251 10.1038/ncb1704

[bib140] Mandal L. Banerjee U. Hartenstein V., 2004 Evidence for a fruit fly hemangioblast and similarities between lymph-gland hematopoiesis in fruit fly and mammal aorta-gonadal-mesonephros mesoderm. Nat. Genet. 36: 1019–1023. 15286786 10.1038/ng1404

[bib141] Mandal L. Martinez-Agosto J. A. Evans C. J. Hartenstein V. Banerjee U., 2007 A Hedgehog- and Antennapedia-dependent niche maintains Drosophila haematopoietic precursors. Nature 446: 320–324. 17361183 10.1038/nature05585 PMC2807630

[bib142] Manley N. R. Selleri L. Brendolan A. Gordon J. Cleary M. L., 2004 Abnormalities of caudal pharyngeal pouch development in Pbx1 knockout mice mimic loss of Hox3 paralogs. Dev. Biol. 276: 301–312. 15581866 10.1016/j.ydbio.2004.08.030

[bib143] Mannstadt M. Bertrand G. Muresan M. Weryha G. Leheup B., 2008 Dominant-negative GCMB mutations cause an autosomal dominant form of hypoparathyroidism. J. Clin. Endocrinol. Metab. 93: 3568–3576. 18583467 10.1210/jc.2007-2167 PMC2567849

[bib144] Mannstadt M. Holick E. Zhao W. Juppner H., 2011 Mutational analysis of GCMB, a parathyroid-specific transcription factor, in parathyroid adenoma of primary hyperparathyroidism. J. Endocrinol. 210: 165–171. 21642377 10.1530/JOE-10-0247 PMC3689587

[bib145] Massimiani M. Vecchione L. Piccirilli D. Spitalieri P. Amati F., 2015 Epidermal growth factor-like domain 7 promotes migration and invasion of human trophoblast cells through activation of MAPK, PI3K and NOTCH signaling pathways. Mol. Hum. Reprod. 21: 435–451. 25667199 10.1093/molehr/gav006 PMC4492406

[bib146] Merscher S. Funke B. Epstein J. A. Heyer J. Puech A., 2001 TBX1 is responsible for cardiovascular defects in velo-cardio-facial/DiGeorge syndrome. Cell 104: 619–629. 11239417 10.1016/s0092-8674(01)00247-1

[bib147] Miller A. A. Bernardoni R. Giangrande A., 1998 Positive autoregulation of the glial promoting factor glide/gcm. EMBO J. 17: 6316–6326. 9799239 10.1093/emboj/17.21.6316 PMC1170956

[bib148] Mitsui T. Narumi S. Inokuchi M. Nagasaki K. Nakazawa M., 2014 Comprehensive next-generation sequencing analyses of hypoparathyroidism: identification of novel GCM2 mutations. J. Clin. Endocrinol. Metab. 99: E2421–E2428. 25137426 10.1210/jc.2014-2174

[bib149] Molkentin J. D., 2000 The zinc finger-containing transcription factors GATA-4, -5, and -6. Ubiquitously expressed regulators of tissue-specific gene expression. J. Biol. Chem. 275: 38949–38952. 11042222 10.1074/jbc.R000029200

[bib150] Mondal B. C. Shim J. Evans C. J. Banerjee U., 2014 Pvr expression regulators in equilibrium signal control and maintenance of Drosophila blood progenitors. eLife 3: e03626. 10.7554/eLife.03626 PMC4185420 25201876

[bib151] Mortimer N. T. Kacsoh B. Z. Keebaugh E. S. Schlenke T. A., 2012 Mgat1-dependent N-glycosylation of membrane components primes Drosophila melanogaster blood cells for the cellular encapsulation response. PLoS Pathog. 8: e1002819. 10.1371/journal.ppat.1002819 PMC3400557 22829770

[bib152] Mukherjee T. Choi I. Banerjee U., 2012 Genetic analysis of fibroblast growth factor signaling in the Drosophila eye. G3 2: 23–28. 22384378 10.1534/g3.111.001495 PMC3276192

[bib153] Muller P. Kuttenkeuler D. Gesellchen V. Zeidler M. P. Boutros M., 2005 Identification of JAK/STAT signalling components by genome-wide RNA interference. Nature 436: 871–875. 16094372 10.1038/nature03869

[bib154] Muratoglu S. Garratt B. Hyman K. Gajewski K. Schulz R. A., 2006 Regulation of Drosophila friend of GATA gene, U-shaped, during hematopoiesis: a direct role for serpent and lozenge. Dev. Biol. 296: 561–579. 16730345 10.1016/j.ydbio.2006.04.455

[bib155] Myllymaki H. Ramet M., 2014 JAK/STAT pathway in Drosophila immunity. Scand. J. Immunol. 79: 377–385. 24673174 10.1111/sji.12170

[bib156] Nabel-Rosen H. Dorevitch N. Reuveny A. Volk T., 1999 The balance between two isoforms of the Drosophila RNA-binding protein how controls tendon cell differentiation. Mol. Cell 4: 573–584. 10549289 10.1016/s1097-2765(00)80208-7

[bib157] Neumuller R. A. Richter C. Fischer A. Novatchkova M. Neumuller K. G., 2011 Genome-wide analysis of self-renewal in Drosophila neural stem cells by transgenic RNAi. Cell Stem Cell 8: 580–593. 21549331 10.1016/j.stem.2011.02.022 PMC3093620

[bib158] O’Brien M. A. Taghert P. H., 1998 A peritracheal neuropeptide system in insects: release of myomodulin-like peptides at ecdysis. J. Exp. Biol. 201: 193–209. 9405303 10.1242/jeb.201.2.193

[bib159] Onel S. F. Rust M. B. Jacob R. Renkawitz-Pohl R., 2014 Tethering membrane fusion: common and different players in myoblasts and at the synapse. J. Neurogenet. 28: 302–315. 24957080 10.3109/01677063.2014.936014 PMC4245166

[bib160] Ozkan E. Carrillo R. A. Eastman C. L. Weiszmann R. Waghray D., 2013 An extracellular interactome of immunoglobulin and LRR proteins reveals receptor-ligand networks. Cell 154: 228–239. 23827685 10.1016/j.cell.2013.06.006 PMC3756661

[bib161] Park D. Shafer O. T. Shepherd S. P. Suh H. Trigg J. S., 2008 The Drosophila basic helix-loop-helix protein DIMMED directly activates PHM, a gene encoding a neuropeptide-amidating enzyme. Mol. Cell. Biol. 28: 410–421. 17967878 10.1128/MCB.01104-07 PMC2223291

[bib162] Park D. Li P. Dani A. Taghert P. H., 2014 Peptidergic cell-specific synaptotagmins in Drosophila: localization to dense-core granules and regulation by the bHLH protein DIMMED. J. Neurosci. 34: 13195–13207. 25253864 10.1523/JNEUROSCI.2075-14.2014 PMC4172809

[bib163] Park S. Y. Eom Y. S. Choi B. Yi H. S. Yu S. H., 2013 Genetic and clinical characteristics of korean patients with isolated hypoparathyroidism: from the Korean hypopara registry study. J. Korean Med. Sci. 28: 1489–1495. 24133354 10.3346/jkms.2013.28.10.1489 PMC3792604

[bib164] Parsons B. Foley E., 2013 The Drosophila platelet-derived growth factor and vascular endothelial growth factor-receptor related (Pvr) protein ligands Pvf2 and Pvf3 control hemocyte viability and invasive migration. J. Biol. Chem. 288: 20173–20183. 23737520 10.1074/jbc.M113.483818 PMC3711285

[bib165] Patel B. N. Van Vactor D. L., 2002 Axon guidance: the cytoplasmic tail. Curr. Opin. Cell Biol. 14: 221–229. 11891122 10.1016/s0955-0674(02)00308-3

[bib166] Perez-Gomez R. Slovakova J. Rives-Quinto N. Krejci A. Carmena A., 2013 A Serrate-Notch-Canoe complex mediates essential interactions between glia and neuroepithelial cells during Drosophila optic lobe development. J. Cell Sci. 126: 4873–4884. 23970418 10.1242/jcs.125617

[bib167] Petersen A. J. Rimkus S. A. Wassarman D. A., 2012 ATM kinase inhibition in glial cells activates the innate immune response and causes neurodegeneration in Drosophila. Proc. Natl. Acad. Sci. USA 109: E656–E664. 22355133 10.1073/pnas.1110470109 PMC3306708

[bib168] Pfarrer C. Weise S. Berisha B. Schams D. Leiser R., 2006 Fibroblast growth factor (FGF)-1, FGF2, FGF7 and FGF receptors are uniformly expressed in trophoblast giant cells during restricted trophoblast invasion in cows. Placenta 27: 758–770. 16129484 10.1016/j.placenta.2005.06.007

[bib169] Pipes G. C. Lin Q. Riley S. E. Goodman C. S., 2001 The Beat generation: a multigene family encoding IgSF proteins related to the Beat axon guidance molecule in Drosophila. Development 128: 4545–4552. 11714679 10.1242/dev.128.22.4545

[bib170] Popkova A. Bernardoni R. Diebold C. Van de Bor V. Schuettengruber B., 2012 Polycomb controls gliogenesis by regulating the transient expression of the Gcm/Glide fate determinant. PLoS Genet. 8: e1003159. 10.1371/journal.pgen.1003159 PMC3531469 23300465

[bib171] Porsch M. Hofmeyer K. Bausenwein B. S. Grimm S. Weber B. H., 1998 Isolation of a Drosophila T-box gene closely related to human TBX1. Gene 212: 237–248. 9611267 10.1016/s0378-1119(98)00180-2

[bib172] Prakash S. McLendon H. M. Dubreuil C. I. Ghose A. Hwa J., 2009 Complex interactions amongst N-cadherin, DLAR, and Liprin-α regulate Drosophila photoreceptor axon targeting. Dev. Biol. 336: 10–19. 19766621 10.1016/j.ydbio.2009.09.016 PMC2783772

[bib173] Prokop A. Martin-Bermudo M. D. Bate M. Brown N. H., 1998 Absence of PS integrins or laminin A affects extracellular adhesion, but not intracellular assembly, of hemiadherens and neuromuscular junctions in Drosophila embryos. Dev. Biol. 196: 58–76. 9527881 10.1006/dbio.1997.8830

[bib174] Ragone G. Bernardoni R. Giangrande A., 2001 A novel mode of asymmetric division identifies the fly neuroglioblast 6–4T. Dev. Biol. 235: 74–85. 11412028 10.1006/dbio.2001.0296

[bib175] Ragone G. Van de Bor V. Sorrentino S. Kammerer M. Galy A., 2003 Transcriptional regulation of glial cell specification. Dev. Biol. 255: 138–150. 12618139 10.1016/s0012-1606(02)00081-7

[bib176] Rajagopalan S. Nicolas E. Vivancos V. Berger J. Dickson B. J., 2000 Crossing the midline: roles and regulation of Robo receptors. Neuron 28: 767–777. 11163265 10.1016/s0896-6273(00)00152-5

[bib177] Rawlings J. S. Rennebeck G. Harrison S. M. Xi R. Harrison D. A., 2004 Two Drosophila suppressors of cytokine signaling (SOCS) differentially regulate JAK and EGFR pathway activities. BMC Cell Biol. 5: 38. 15488148 10.1186/1471-2121-5-38 PMC526380

[bib178] Rayon T. Menchero S. Nieto A. Xenopoulos P. Crespo M., 2014 Notch and hippo converge on Cdx2 to specify the trophectoderm lineage in the mouse blastocyst. Dev. Cell 30: 410–422. 25127056 10.1016/j.devcel.2014.06.019 PMC4146744

[bib179] Reeh K. A. Cardenas K. T. Bain V. E. Liu Z. Laurent M., 2014 Ectopic TBX1 suppresses thymic epithelial cell differentiation and proliferation during thymus organogenesis. Development 141: 2950–2958. 25053428 10.1242/dev.111641 PMC4197657

[bib180] Ren C. Finkel S. E. Tower J., 2009 Conditional inhibition of autophagy genes in adult Drosophila impairs immunity without compromising longevity. Exp. Gerontol. 44: 228–235. 18955126 10.1016/j.exger.2008.10.002 PMC2664319

[bib181] Rizki M. T. M., 1957 Alterations in the haemocyte population of Drosophila melanogaster. J. Morphol. 100: 437–458.

[bib182] Rudolph K. M. Liaw G. J. Daniel A. Green P. Courey A. J., 1997 Complex regulatory region mediating tailless expression in early embryonic patterning and brain development. Development 124: 4297–4308. 9334278 10.1242/dev.124.21.4297

[bib183] Sarraf-Zadeh L. Christen S. Sauer U. Cognigni P. Miguel-Aliaga I., 2013 Local requirement of the Drosophila insulin binding protein imp-L2 in coordinating developmental progression with nutritional conditions. Dev. Biol. 381: 97–106. 23773803 10.1016/j.ydbio.2013.06.008

[bib184] Schaaf C. A. Misulovin Z. Gause M. Koenig A. Dorsett D., 2013 The Drosophila enhancer of split gene complex: architecture and coordinate regulation by notch, cohesin, and polycomb group proteins. G3 3: 1785–1794. 23979932 10.1534/g3.113.007534 PMC3789803

[bib185] Schmucker D. Clemens J. C. Shu H. Worby C. A. Xiao J., 2000 Drosophila Dscam is an axon guidance receptor exhibiting extraordinary molecular diversity. Cell 101: 671–684. 10892653 10.1016/s0092-8674(00)80878-8

[bib186] Schreiber J. Sock E. Wegner M., 1997 The regulator of early gliogenesis glial cells missing is a transcription factor with a novel type of DNA-binding domain. Proc. Natl. Acad. Sci. USA 94: 4739–4744. 9114061 10.1073/pnas.94.9.4739 PMC20794

[bib187] Schreiber J. Enderich J. Wegner M., 1998 Structural requirements for DNA binding of GCM proteins. Nucleic Acids Res. 26: 2337–2343. 9580683 10.1093/nar/26.10.2337 PMC147556

[bib188] Schwabe T. Gontang A. C. Clandinin T. R., 2009 More than just glue: the diverse roles of cell adhesion molecules in the Drosophila nervous system. Cell Adhes. Migr. 3: 36–42. 10.4161/cam.3.1.6918 PMC2675147 19372748

[bib189] Schwanbeck R. Martini S. Bernoth K. Just U., 2011 The Notch signaling pathway: molecular basis of cell context dependency. Eur. J. Cell Biol. 90: 572–581. 21126799 10.1016/j.ejcb.2010.10.004

[bib190] Seeger M. Tear G. Ferres-Marco D. Goodman C. S., 1993 Mutations affecting growth cone guidance in Drosophila: genes necessary for guidance toward or away from the midline. Neuron 10: 409–426. 8461134 10.1016/0896-6273(93)90330-t

[bib191] Sen M. Ghosht G., 2008 Transcriptional outcome of Wnt-Frizzled signal transduction in inflammation: Evolving concepts. J. Immunol. 181: 4441–4445. 18802045 10.4049/jimmunol.181.7.4441

[bib192] Shandala T. Takizawa K. Saint R., 2003 The dead ringer/retained transcriptional regulatory gene is required for positioning of the longitudinal glia in the Drosophila embryonic CNS. Development 130: 1505–1513. 12620977 10.1242/dev.00377

[bib193] Shimizu K. Chiba S. Hosoya N. Kumano K. Saito T., 2000 Binding of Delta1, Jagged1, and Jagged2 to Notch2 rapidly induces cleavage, nuclear translocation, and hyperphosphorylation of Notch2. Mol. Cell. Biol. 20: 6913–6922. 10958687 10.1128/mcb.20.18.6913-6922.2000 PMC88767

[bib194] Shishido E. Higashijima S. Emori Y. Saigo K., 1993 2 Fgf-receptor homologs of Drosophila: one is expressed in mesodermal primordium in early embryos. Development 117: 751–761. 8330538 10.1242/dev.117.2.751

[bib195] Shishido E. Ono N. Kojima T. Saigo K., 1997 Requirements of DFR1/Heartless, a mesoderm-specific Drosophila FGF-receptor, for the formation of heart, visceral and somatic muscles, and ensheathing of longitudinal axon tracts in CNS. Development 124: 2119–2128. 9187139 10.1242/dev.124.11.2119

[bib196] Siekhaus D. Haesemeyer M. Moffitt O. Lehmann R., 2010 RhoL controls invasion and Rap1 localization during immune cell transmigration in Drosophila. Nat. Cell Biol. 12: 605–610. 20495554 10.1038/ncb2063 PMC3006444

[bib197] Siepel A. Bejerano G. Pedersen J. S. Hinrichs A. S. Hou M., 2005 Evolutionarily conserved elements in vertebrate, insect, worm, and yeast genomes. Genome Res. 15: 1034–1050. 16024819 10.1101/gr.3715005 PMC1182216

[bib198] Song J. Tanouye M. A., 2006 Seizure suppression by shakB2, a gap junction mutation in Drosophila. J. Neurophysiol. 95: 627–635. 16192342 10.1152/jn.01059.2004

[bib199] Song J. B. Wu L. L. Chen Z. Kohanski R. A. Pick L., 2003 Axons guided by insulin receptor in Drosophila visual system. Science 300: 502–505. 12702880 10.1126/science.1081203

[bib200] Song S. Ge Q. Wang J. Chen H. Tang S., 2011 TRIM-9 functions in the UNC-6/UNC-40 pathway to regulate ventral guidance. J. Genet. Genomics 38: 1–11. 21338947 10.1016/j.jcg.2010.12.004

[bib201] Sorrentino R. P. Tokusumi T. Schulz R. A., 2007 The Friend of GATA protein U-shaped functions as a hematopoietic tumor suppressor in Drosophila. Dev. Biol. 311: 311–323. 17936744 10.1016/j.ydbio.2007.08.011

[bib202] Soustelle L. Giangrande A., 2007 Novel gcm-dependent lineages in the postembryonic nervous system of Drosophila melanogaster. Dev. Dyn. 236: 2101–2108. 17654713 10.1002/dvdy.21232

[bib203] Soustelle L. Jacques C. Altenhein B. Technau G. M. Volk T., 2004 Terminal tendon cell differentiation requires the glide/gcm complex. Development 131: 4521–4532. 15342477 10.1242/dev.01290

[bib204] Soustelle L. Trousse F. Jacques C. Ceron J. Cochard P., 2007 Neurogenic role of Gcm transcription factors is conserved in chicken spinal cord. Development 134: 625–634. 17215311 10.1242/dev.02750

[bib205] Southall T. D. Brand A. H., 2009 Neural stem cell transcriptional networks highlight genes essential for nervous system development. EMBO J. 28: 3799–3807. 19851284 10.1038/emboj.2009.309 PMC2770102

[bib206] Sprecher S. G. Hirth F., 2006 Expression and function of the columnar patterning gene msh in late embryonic brain development of Drosophila. Dev. Dyn. 235: 2920–2929. 16929521 10.1002/dvdy.20936

[bib207] Starz-Gaiano M. Melani M. Wang X. B. Meinhardt H. Montell D. J., 2008 Feedback inhibition of JAK/STAT signaling by apontic is required to limit an invasive cell population. Dev. Cell 14: 726–738. 18477455 10.1016/j.devcel.2008.03.005

[bib208] Stec W. Vidal O. Zeidler M. P., 2013 Drosophila SOCS36E negatively regulates JAK/STAT pathway signaling via two separable mechanisms. Mol. Biol. Cell 24: 3000–3009. 23885117 10.1091/mbc.E13-05-0275 PMC3771960

[bib209] Steigemann P. Molitor A. Fellert S. Jackle H. Vorbruggen G., 2004 Heparan sulfate proteoglycan syndecan promotes axonal and myotube guidance by slit/Robo signaling. Curr. Biol. 14: 225–230. 14761655 10.1016/j.cub.2004.01.006

[bib210] Stofanko M. Kwon S. Y. Badenhorst P., 2010 Lineage tracing of lamellocytes demonstrates Drosophila macrophage plasticity. PLoS One 5: e14051. 10.1371/journal.pone.0014051 PMC2988793 21124962

[bib211] Stork T. Sheehan A. Tasdemir-Yilmaz O. E. Freeman M. R., 2014 Neuron-glia interactions through the Heartless FGF receptor signaling pathway mediate morphogenesis of Drosophila astrocytes. Neuron 83: 388–403. 25033182 10.1016/j.neuron.2014.06.026 PMC4124900

[bib212] Strigini M. Cantera R. Morin X. Bastiani M. J. Bate M., 2006 The IgLON protein Lachesin is required for the blood-brain barrier in Drosophila. Mol. Cell. Neurosci. 32: 91–101. 16682215 10.1016/j.mcn.2006.03.001

[bib213] Stroschein-Stevenson S. L. Foley E. O’Farrell P. H. Johnson A. D., 2006 Identification of Drosophila gene products required for phagocytosis of Candida albicans. PLoS Biol. 4: e4. 10.1371/journal.pbio.0040004 PMC1310651 16336044

[bib214] Sugie A. Umetsu D. Yasugi T. Fischbach K. F. Tabata T., 2010 Recognition of pre- and postsynaptic neurons via nephrin/NEPH1 homologs is a basis for the formation of the Drosophila retinotopic map. Development 137: 3303–3313. 20724453 10.1242/dev.047332

[bib215] Sun Q. Schindelholz B. Knirr M. Schmid A. Zinn K., 2001 Complex genetic interactions among four receptor tyrosine phosphatases regulate axon guidance in Drosophila. Mol. Cell. Neurosci. 17: 274–291. 11178866 10.1006/mcne.2000.0939

[bib216] Tao Y. Wang J. Tokusumi T. Gajewski K. Schulz R. A., 2007 Requirement of the LIM homeodomain transcription factor tailup for normal heart and hematopoietic organ formation in Drosophila melanogaster. Mol. Cell. Biol. 27: 3962–3969. 17371844 10.1128/MCB.00093-07 PMC1900034

[bib217] Thomas G. B. van Meyel D. J., 2007 The glycosyltransferase Fringe promotes Delta-Notch signaling between neurons and glia, and is required for subtype-specific glial gene expression. Development 134: 591–600. 17215308 10.1242/dev.02754

[bib218] Tomancak P. Beaton A. Weiszmann R. Kwan E. Shu S., 2002 Systematic determination of patterns of gene expression during Drosophila embryogenesis. Genome Biol. 3: RESEARCH0088. 10.1186/gb-2002-3-12-research0088 PMC151190 12537577

[bib219] Tomancak P. Berman B. P. Beaton A. Weiszmann R. Kwan E., 2007 Global analysis of patterns of gene expression during Drosophila embryogenesis. Genome Biol. 8: R145. 17645804 10.1186/gb-2007-8-7-r145 PMC2323238

[bib220] Udolph G. Rath P. Chia W., 2001 A requirement for Notch in the genesis of a subset of glial cells in the Drosophila embryonic central nervous system which arise through asymmetric divisions. Development 128: 1457–1466. 11262244 10.1242/dev.128.8.1457

[bib221] Uhlen M. Fagerberg L. Hallstrom B. M. Lindskog C. Oksvold P., 2015 Proteomics. Tissue-based map of the human proteome. Science 347: 1260419. 25613900 10.1126/science.1260419

[bib222] Umesono Y. Hiromi Y. Hotta Y., 2002 Context-dependent utilization of Notch activity in Drosophila glial determination. Development 129: 2391–2399. 11973271 10.1242/dev.129.10.2391

[bib223] Umetsu D. Murakami S. Sato M. Tabata T., 2006 The highly ordered assembly of retinal axons and their synaptic partners is regulated by Hedgehog/Single-minded in the Drosophila visual system. Development 133: 791–800. 16439478 10.1242/dev.02253

[bib224] Van de Bor V. Giangrande A., 2001 Notch signaling represses the glial fate in fly PNS. Development 128: 1381–1390. 11262238 10.1242/dev.128.8.1381

[bib225] van Steensel B. Henikoff S., 2000 Identification of in vivo DNA targets of chromatin proteins using tethered dam methyltransferase. Nat. Biotechnol. 18: 424–428. 10748524 10.1038/74487

[bib226] van Steensel B. Delrow J. Henikoff S., 2001 Chromatin profiling using targeted DNA adenine methyltransferase. Nat. Genet. 27: 304–308. 11242113 10.1038/85871

[bib227] Veenstra J. A., 2009 Peptidergic paracrine and endocrine cells in the midgut of the fruit fly maggot. Cell Tissue Res. 336: 309–323. 19319573 10.1007/s00441-009-0769-y

[bib228] Veenstra J. A. Agricola H. J. Sellami A., 2008 Regulatory peptides in fruit fly midgut. Cell Tissue Res. 334: 499–516. 18972134 10.1007/s00441-008-0708-3

[bib229] Vincent S. Vonesch J. L. Giangrande A., 1996 Glide directs glial fate commitment and cell fate switch between neurones and glia. Development 122: 131–139. 8565824 10.1242/dev.122.1.131

[bib230] von Hilchen C. M. Hein I. Technau G. M. Altenhein B., 2010 Netrins guide migration of distinct glial cells in the Drosophila embryo. Development 137: 1251–1262. 20223758 10.1242/dev.042853

[bib231] Waltzer L. Gobert V. Osman D. Haenlin M., 2010 Transcription factor interplay during Drosophila haematopoiesis. Int. J. Dev. Biol. 54: 1107–1115. 20711988 10.1387/ijdb.093054lw

[bib232] Wang H. Chen X. He T. Zhou Y. Luo H., 2013 Evidence for tissue-specific Jak/STAT target genes in Drosophila optic lobe development. Genetics 195: 1291–1306. 24077308 10.1534/genetics.113.155945 PMC3832274

[bib233] Wang W. Liu W. Wang Y. Zhou L. Tang X., 2011 Notch signaling regulates neuroepithelial stem cell maintenance and neuroblast formation in Drosophila optic lobe development. Dev. Biol. 350: 414–428. 21146517 10.1016/j.ydbio.2010.12.002

[bib234] Wang Z. Berkey C. D. Watnick P. I., 2012 The Drosophila protein mustard tailors the innate immune response activated by the immune deficiency pathway. J. Immunol. 188: 3993–4000. 22427641 10.4049/jimmunol.1103301 PMC3324637

[bib235] Watson F. L. Puttmann-Holgado R. Thomas F. Lamar D. L. Hughes M., 2005 Extensive diversity of Ig-superfamily proteins in the immune system of insects. Science 309: 1874–1878. 16109846 10.1126/science.1116887

[bib236] Wayburn B. Volk T., 2009 LRT, a tendon-specific leucine-rich repeat protein, promotes muscle-tendon targeting through its interaction with Robo. Development 136: 3607–3615. 19793885 10.1242/dev.040329

[bib237] Xiong W. C. Okano H. Patel N. H. Blendy J. A. Montell C., 1994 repo encodes a glial-specific homeo domain protein required in the Drosophila nervous system. Genes Dev. 8: 981–994. 7926782 10.1101/gad.8.8.981

[bib238] Yano T. Mita S. Ohmori H. Oshima Y. Fujimoto Y., 2008 Autophagic control of listeria through intracellular innate immune recognition in drosophila. Nat. Immunol. 9: 908–916. 18604211 10.1038/ni.1634 PMC2562576

[bib239] Yarnitzky T. Min L. Volk T., 1997 The Drosophila neuregulin homolog Vein mediates inductive interactions between myotubes and their epidermal attachment cells. Genes Dev. 11: 2691–2700. 9334331 10.1101/gad.11.20.2691 PMC316610

[bib240] Yasugi T. Sugie A. Umetsu D. Tabata T., 2010 Coordinated sequential action of EGFR and Notch signaling pathways regulates proneural wave progression in the Drosophila optic lobe. Development 137: 3193–3203. 20724446 10.1242/dev.048058

[bib241] Yi H. S. Eom Y. S. Park Ie B. Lee S. Hong S., 2012 Identification and characterization of C106R, a novel mutation in the DNA-binding domain of GCMB, in a family with autosomal-dominant hypoparathyroidism. Clin. Endocrinol. 76: 625–633. 10.1111/j.1365-2265.2011.04256.x PMC3701386 22066718

[bib242] Yoshida S. Soustelle L. Giangrande A. Umetsu D. Murakami S., 2005 DPP signaling controls development of the lamina glia required for retinal axon targeting in the visual system of Drosophila. Development 132: 4587–4598. 16176948 10.1242/dev.02040

[bib243] Yu C. Shen K. Lin M. Chen P. Lin C., 2002 GCMa regulates the syncytin-mediated trophoblastic fusion. J. Biol. Chem. 277: 50062–50068. 12397062 10.1074/jbc.M209316200

[bib244] Yuan Q. Xiang Y. Yan Z. Han C. Jan L. Y., 2011 Light-induced structural and functional plasticity in Drosophila larval visual system. Science 333: 1458–1462. 21903815 10.1126/science.1207121 PMC4114502

[bib245] Yuasa Y. Okabe M. Yoshikawa S. Tabuchi K. Xiong W. C., 2003 Drosophila homeodomain protein REPO controls glial differentiation by cooperating with ETS and BTB transcription factors. Development 130: 2419–2428. 12702656 10.1242/dev.00468

[bib246] Zacharioudaki E. Magadi S. S. Delidakis C., 2012 bHLH-O proteins are crucial for Drosophila neuroblast self-renewal and mediate Notch-induced overproliferation. Development 139: 1258–1269. 22357926 10.1242/dev.071779

[bib247] Zanet J. Stramer B. Millard T. Martin P. Payre F., 2009 Fascin is required for blood cell migration during Drosophila embryogenesis. Development 136: 2557–2565. 19592575 10.1242/dev.036517

[bib248] Zaytouni T. Efimenko E. E. Tevosian S. G., 2011 GATA transcription factors in the developing reproductive system. Adv. Genet. 76: 93–134. 22099693 10.1016/B978-0-12-386481-9.00004-3

[bib249] Zeev-Ben-Mordehai T. Paz A. Peleg Y. Toker L. Wolf S. G., 2009 Amalgam, an axon guidance Drosophila adhesion protein belonging to the immunoglobulin superfamily: over-expression, purification and biophysical characterization. Protein Expr. Purif. 63: 147–157. 18938249 10.1016/j.pep.2008.09.019

[bib250] Zettervall C. J. Anderl I. Williams M. J. Palmer R. Kurucz E., 2004 A directed screen for genes involved in Drosophila blood cell activation. Proc. Natl. Acad. Sci. USA 101: 14192–14197. 15381778 10.1073/pnas.0403789101 PMC521135

[bib251] Zhang J. Carthew R. W., 1998 Interactions between Wingless and DFz2 during Drosophila wing development. Development 125: 3075–3085. 9671581 10.1242/dev.125.16.3075

[bib252] Zhao W. X. Lin J. H., 2012 Notch signaling pathway and human placenta. Int. J. Med. Sci. 9: 447–452. 10.7150/ijms.4593 PMC3410364 22859905

[bib253] Zhu F. Zhang X. B., 2013 The Wnt signaling pathway is involved in the regulation of phagocytosis of virus in Drosophila. Sci. Rep. 3: 2068. 23797713 10.1038/srep02069 PMC3691566

